# Oxygen self-sufficient NIR-activatable liposomes for tumor hypoxia regulation and photodynamic therapy†
†Electronic supplementary information (ESI) available. See DOI: 10.1039/c9sc03161h


**DOI:** 10.1039/c9sc03161h

**Published:** 2019-08-08

**Authors:** Qi Yu, Tianci Huang, Chao Liu, Menglong Zhao, Mingjuan Xie, Guo Li, Shujuan Liu, Wei Huang, Qiang Zhao

**Affiliations:** a Key Laboratory for Organic Electronics and Information Displays , Jiangsu Key Laboratory for Biosensors , Institute of Advanced Materials (IAM) , Nanjing University of Posts and Telecommunications (NUPT) , Nanjing 210023 , P. R. China . Email: iamqzhao@njupt.edu.cn; b Shaanxi Institute of Flexible Electronics (SIFE) , Northwestern Polytechnical University (NPU) , Xi'an 710072 , Shaanxi , P. R. China . Email: provost@nwpu.edu.cn

## Abstract

The inherent hypoxic environment in tumors severely resists the efficacy of photodynamic therapy. To address this problem, herein, the strategy of using oxygen self-sufficient liposomes (denoted as CaO_2_/**B1**/NH_4_HCO_3_ lipo), which contained aza-BODIPY dye (**B1**) and CaO_2_ nanoparticles in the hydrophobic layer and NH_4_HCO_3_ in the hydrophilic cavity, was presented to overcome hypoxia-associated photodynamic resistance. Under near-infrared (NIR) irradiation, NIR-absorbable **B1** was activated to induce hyperthermia and further triggered the decomposition of NH_4_HCO_3_. Subsequently, with the aid of NH_4_HCO_3_ and CaO_2_ nanoparticles, oxygen was rapidly and self-sufficiently generated, during which clean by-products were produced. Furthermore, the increased amount of oxygen promoted the singlet oxygen production in the presence of **B1**, which served as a photosensitizer because of the heavy atom effect. The oxygen self-sufficient system improved the anticancer efficiency and alleviated the hypoxic environment *in vivo*, which demonstrated a valuable attempt to regulate intratumoral hypoxia and overcome the limitation of current photodynamic therapy systems. To our knowledge, this highlights the first example of using NIR light to activate CaO_2_ nanoparticle-containing liposomes for the modulation of the hypoxic environment in tumors.

## Abstract 2

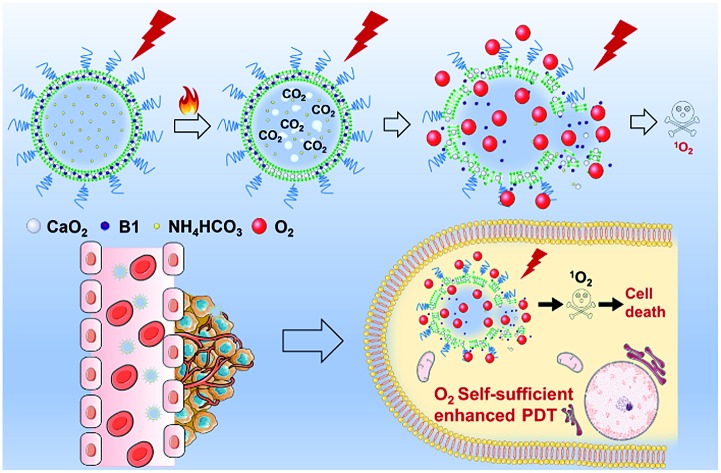
We have presented oxygen self-sufficient near infrared-activatable liposomes to overcome hypoxia-associated photodynamic resistance.

## Introduction

Hypoxia, one of the most remarkable features in tumorous microenvironments, has been demonstrated to be associated with many aspects of the activities of tumors, such as altered metabolism, unstabilized genome and metastasis.^1^–^7^ Moreover, it results in the limited response and resistance of treatments using antitumor drugs and radioactive reagents.^8^–^11^ Especially, the absence of adequate oxygen in tumors has been proved to largely influence the existing photodynamic therapy (PDT) systems, which mainly rely on a photosensitizing reagent to undergo the type II pathway to produce cytotoxic singlet oxygen (^1^O_2_) under photoexcitation.^12^–^20^ Moreover, the hypoxic environment can be further aggravated because of oxygen consumption during the PDT process.^21^,^22^ To overcome the intratumoral hypoxia, many oxygen-generating materials have been developed to supply oxygen, such as oxygen loading perfluorocarbon, intracellular hydrogen peroxide (H_2_O_2_) catalytic catalase and manganese dioxide (MnO_2_).^23^–^32^ Although the reported oxygen-generating materials have shown some promise, the amount of enriched oxygen is still difficult to improve because of the naturally limited effects and low endogenous H_2_O_2_ concentration.^33^,^34^ In this regard, it is highly desirable to design a new generation of anti-hypoxia nanocarriers with enhanced and self-sufficient oxygen supplementation.

Calcium peroxide (CaO_2_) has been employed as a kind of oxygen-generating material.^35^ Different from other alkaline earth metal peroxides, it can slowly release oxygen in humid air and has been widely used in soil treatment and water disinfection.^36^–^39^ Inspired by this, CaO_2_ has recently been introduced as an implantable oxygen-generating material to overcome the hypoxia-induced restriction towards chemotherapy in cells and tissues.^40^ The self-sufficient oxygen supplementation made CaO_2_ a potential biomaterial that can synergistically work with a photosensitizing reagent and improve PDT efficiency, but the oversized structure suffered from limited penetration in the tumor tissue and the difficulty of clearance from the body. An alternative strategy has emerged to explore the small-sized CaO_2_ nanoparticles as oxygen-generating materials.^41^ However the slow and passive oxygen release from CaO_2_ nanoparticles compromised its efficacy after approaching the tumor tissues. Additionally, the gradually increased concentration of alkaline metabolites during oxygen generation results in potential long-term side effects on healthy tissues and organs.

**Fig. 1:**
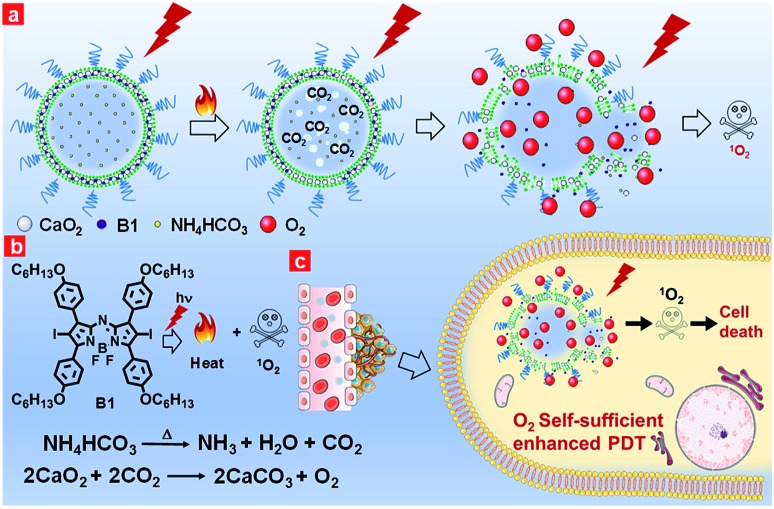
(a) NIR-regulated generation of oxygen in CaO_2_/**B1**/NH_4_HCO_3_ lipo and enhancement of ^1^O_2_; (b) the mechanism of oxygen generation in CaO_2_/**B1**/NH_4_HCO_3_ lipo; (c) *in vivo* the oxygen self-sufficient CaO_2_/**B1**/NH_4_HCO_3_ lipo enhanced PDT and killed the cancer cells.

Keeping the aforementioned issues in mind, herein, we proposed oxygen self-sufficient liposomes (CaO_2_/**B1**/NH_4_HCO_3_ lipo), which consisted of hydrophobic halogenated aza-BODIPY dye (**B1**), oxygen-generating CaO_2_ nanoparticles, and hydrophilic thermoresponsive ammonium bicarbonate (NH_4_HCO_3_) to regulate the hypoxic tumor microenvironment and overcome hypoxia-induced photodynamic resistance (Fig. 1a). In the liposomes, halogenated **B1** was regarded as not only a potential photosensitizer through the preferable singlet-to-triplet transition but also a good organic photothermal agent because of strong near-infrared (NIR) absorption.^42^,^43^ Under irradiation, **B1** generated heat and triggered the decomposition of NH_4_HCO_3_, thereby resulting in the generation of CO_2_ bubbles. Thus, with the aid of **B1** and NH_4_HCO_3_, CaO_2_ nanoparticles were induced to rapidly release oxygen by reaction with CO_2_. Additionally, the clean metabolite produced during the reaction reduced the toxicity to the body. Taking advantage of the enhanced self-sufficient oxygen, the hypoxia environment could be regulated and the photodynamic resistance in the tumors could be overcome. In *in vivo* environments, the liposomes were observed to accumulate in tumorous tissues through tail intravenous injection, which led to improved PDT efficiency under NIR irradiation. The relieved intratumoral hypoxia environment was demonstrated through immunofluorescence staining of hypoxia-associated proteins.

## Results and discussion

In this work, we selected the polyethylene glycol (PEG) shelled liposome system as the nanocarrier owing to its good biocompatibility, enhanced tumor permeation and retention, and efficient loading capacity. The PEGylated liposomes included three elements, namely CaO_2_ nanoparticles, NH_4_HCO_3_ and halogenated aza-BODIPY (**B1**). CaO_2_ nanoparticles served as the resource of oxygen supplementation. The thermoresponsive molecule NH_4_HCO_3_, which can produce CO_2_ when temperature reaches 40 °C,^44^ was employed as the stimulus to trigger the rapid release of oxygen from CaO_2_ nanoparticles. NIR dye **B1** served as both a photothermal agent and a photosensitizer for PDT owing to the heavy atom effect.^45^

**Fig. 2:**
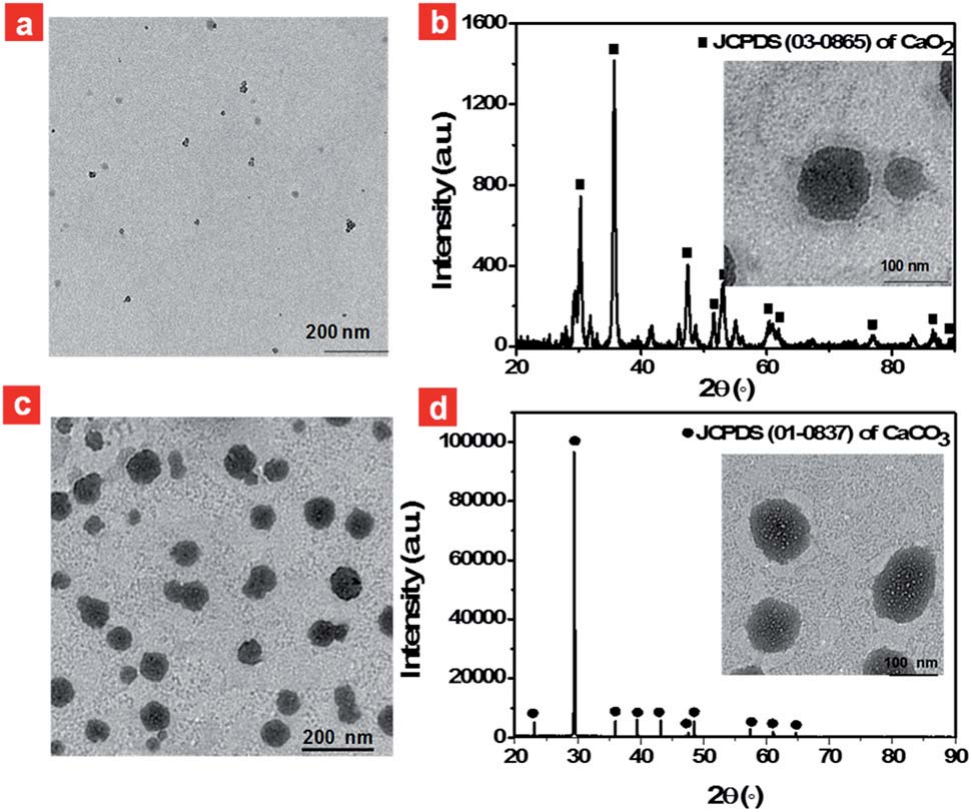
TEM images of CaO_2_ nanoparticles (a) and CaO_2_/**B1**/NH_4_HCO_3_ lipo (c); XRD patterns of CaO_2_/**B1**/NH_4_HCO_3_ lipo before (b) and after heating to 45 °C (d); insets show TEM images of CaO_2_/**B1**/NH_4_HCO_3_ lipo before and after heating to 45 °C, respectively.

Firstly, CaO_2_ nanoparticles were prepared *via* the reaction of the calcium salt with H_2_O_2_ in the presence of ammonia. TEM images of CaO_2_ nanoparticles clearly showed a spherical structure and the average diameter was in the range of 5–15 nm (Fig. 2a). The dynamic light scattering (DLS) result revealed that CaO_2_ nanoparticles were well dispersed in aqueous solution and had an average diameter of 37.5 nm (Fig. S1a†). The X-ray diffraction (XRD) pattern of the prepared CaO_2_ nanoparticles displayed the presence of peaks at 2*θ* = 30.2°, 35.6°, 47.3°, 51.6°, 53.1°, 60.4° (Fig. 2b), which were in accordance with the 2*θ* position of standard CaO_2_ (JCPDS 03-0865). Then hydrophobic **B1** and CaO_2_ nanoparticles, together with hydrophilic NH_4_HCO_3_, were respectively encapsulated into the lipid bilayers and the aqueous cavity of the polyethylene glycol shelled liposomes through the lipid film hydration technique. NH_4_HCO_3_, CaO_2_/NH_4_HCO_3_, CaO_2_/**B1** and CaO_2_/NH_4_HCO_3_ lipo were also prepared as the control. The detailed synthetic procedures are described in the ESI.†

Typically, CaO_2_/**B1**/NH_4_HCO_3_ lipo exhibited a spherical structure with a size of about 80 nm (Fig. 2c), which was optimized using different sonication powers (Fig. S2†). The DLS measurement gave a hydrodynamic size of 124.1 nm (Fig. S1b†). The successful assembly of CaO_2_ nanoparticles was characterized by inductively coupled plasma-atomic emission spectrometry with a 37.0% weight ratio in the liposomes. Bands located at 765 nm and 823 nm, respectively, in the absorption and photoluminescence spectra of CaO_2_/**B1**/NH_4_HCO_3_ lipo (Fig. S3a and b†) were observed and could be ascribed to the **B1** molecule. The NIR absorption and emission guaranteed that the liposomes could be regarded as a potential imaging reagent *in vivo*. The loading weight of **B1** in the liposomes was estimated to be 17.7%. We fixed the amount of CaO_2_ nanoparticles and **B1** in the lipid bilayers of the liposomes to balance the capacity for oxygen generation and photothermal effects. Additionally, bright-field images in the microscopy showed the formation of bubbles in the solution containing NH_4_HCO_3_ lipo at 45 °C (Fig. S4†), while no bubbles were observed at 37 °C, suggesting that thermoresponsive NH_4_HCO_3_ was capable of well trapping in the liposome structure and underwent decomposition to produce CO_2_.

After successful incorporation of these elements into the displayed liposome structure, we clarified the reaction that occurred in CaO_2_/**B1**/NH_4_HCO_3_ lipo under local hyperthermia. The solution containing CaO_2_/**B1**/NH_4_HCO_3_ lipo was kept at 45 °C for 30 min and added dropwise onto a copper grid for TEM analysis. The Fig. 2b inset displays the images of CaO_2_/**B1**/NH_4_HCO_3_ lipo after heating. Small bubbles appeared on the surface of CaO_2_/**B1**/NH_4_HCO_3_ lipo. These liposomes were observed to swell and their diameters reached about 100 nm. Additionally, the position of XRD peaks from CaO_2_/**B1**/NH_4_HCO_3_ lipo under local hyperthermia corresponded to the standard CaCO_3_ (JCPDS 01-0837). The phenomenon suggested that the gas produced from the liposomes was originated from the reaction between CaO_2_ nanoparticles and CO_2_ from the decomposition of NH_4_HCO_3_.

**Fig. 3:**
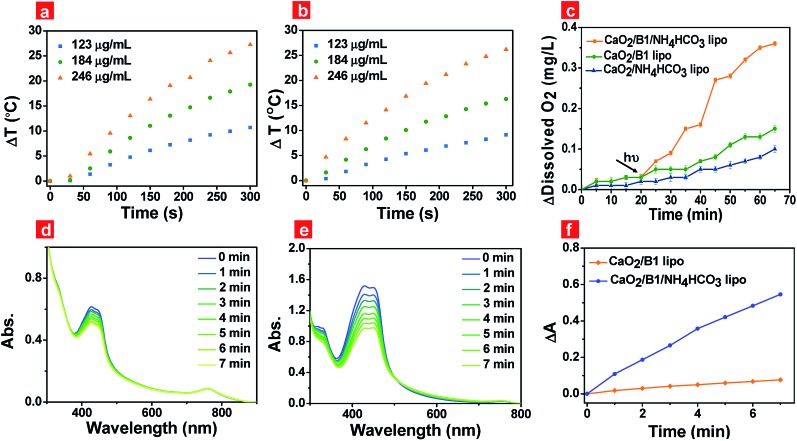
Temperature changes of CaO_2_/**B1** lipo (a) and CaO_2_/**B1**/NH_4_HCO_3_ lipo (b) at a **B1** concentration of 20, 30 and 40 μM under different irradiation times; (c) oxygen generation of CaO_2_/**B1** lipo, CaO_2_/**B1**/NH_4_HCO_3_ lipo and CaO_2_/NH_4_HCO_3_ lipo (184 μg mL^–1^) without and with irradiation; absorption spectra of a mixture containing DPBF and CaO_2_/**B1** lipo (d), CaO_2_/**B1**/NH_4_HCO_3_ lipo (e) (184 μg mL^–1^) at different irradiation times in the hypoxia environment. (f) Δ*A* of DPBF at 427 nm which is obtained from figures (d) and (e). Δ*A* = *A*_t_ – *A*_0_, where *A*_t_ is the absorbance of DPBF at 427 nm at different illumination times and *A*_0_ is the absorption without irradiation. The excitation wavelength of irradiation is 730 nm. The power density is 500 mW cm^–2^.

Photothermal effects of the liposomes were the key factor to guarantee the rapid generation of oxygen. Thus we evaluated the photothermal effects of the prepared liposomes under 730 nm irradiation at 500 mW cm^–2^ through a thermal infrared imager (Fig. S5a†). Liposomes containing **B1** units were capable of elevating the solution temperature (Fig. 3a and b) when the irradiation time was prolonged, while liposomes in the absence of **B1** units were not (Fig. S5b†). The temperature elevation reached up to about 27.3 °C and 26.2 °C for CaO_2_/**B1**/NH_4_HCO_3_ lipo and CaO_2_/**B1** lipo at a **B1** loading concentration of 40 μM, respectively, after the irradiation time reached 300 s, indicating that **B1** played an effective role in the photothermal effects.

To demonstrate our hypothesis about the rapid and enhanced oxygen generation of CaO_2_/**B1**/NH_4_HCO_3_ lipo through photothermal effects, the oxygen generation was tested through a portable dissolved oxygen meter (Fig. 3c). Without irradiation, the concentration of oxygen released from CaO_2_/**B1**/NH_4_HCO_3_, CaO_2_/NH_4_HCO_3_ and CaO_2_/**B1** lipo was observed to slowly increase for 20 min. As expected, irradiation (730 nm, 500 mW cm^–2^) triggered the rapid generation of oxygen in the buffer solution containing CaO_2_/**B1**/NH_4_HCO_3_ lipo. Compared to CaO_2_/**B1**/NH_4_HCO_3_ lipo, CaO_2_/NH_4_HCO_3_ lipo and CaO_2_/**B1** lipo exhibited a relatively small amount of oxygen generation, which was attributed to the sustained hydrolysis of CaO_2_ nanoparticles. These results demonstrated that CaO_2_ nanoparticles in the liposomes efficiently and rapidly supplied oxygen with the aid of photothermal effects from **B1** and thermal reaction from NH_4_HCO_3_. Additionally, the pH values of the aqueous solution, which contained CaO_2_/**B1**/NH_4_HCO_3_ lipo with different concentrations, were examined to range between 7.1 and 8.3 under irradiation for 20 min (Fig. S6†), indicating small effects of metabolites on pH values. The slight increase of the initial pH in the solution containing the concentrated liposomes could be ascribed to the increased opportunity of reaction between CaO_2_ nanoparticles and water.

To pave the way for the utilization of CaO_2_/**B1**/NH_4_HCO_3_ lipo in hypoxic PDT, the light-induced ^1^O_2_ release was assessed using 1,3-diphenylisobenzofuran (DPBF), a ^1^O_2_ indicator, in air and the hypoxia environment that was built by bubbling with nitrogen gas. ROS generation experiments were performed at 37 °C to imitate the *in vivo* environment. In air, a decreased absorbance of DPBF at 427 nm was observed in the liposome containing **B1** units under irradiation (Fig. S7a and b†), suggesting that **B1** served as a photosensitizer to release ^1^O_2_ through the preferable singlet-to-triplet transition. Rapid ^1^O_2_ generation was observed in the group of CaO_2_/**B1**/NH_4_HCO_3_ lipo (Fig. S7c†), indicative of the important role of the oxygen supplement from the reaction between CaO_2_ nanoparticles and NH_4_HCO_3_. The light-induced oxygen supplementation enhanced the probability of energy transfer between excited **B1** and oxygen, leading to an increased amount of ^1^O_2_ released. In the hypoxia environment, ^1^O_2_ release was largely limited from CaO_2_/**B1** lipo (Fig. 3d), while ^1^O_2_ was observed to be more efficiently and rapidly released from CaO_2_/**B1**/NH_4_HCO_3_ lipo (Fig. 3e). The absorbance changes illustrated in Fig. 3f displayed the enhanced amount of ^1^O_2_ released in the hypoxia environment. The results showed the potential utilization of CaO_2_/**B1**/NH_4_HCO_3_ lipo in the hypoxia environment.

**Fig. 4:**
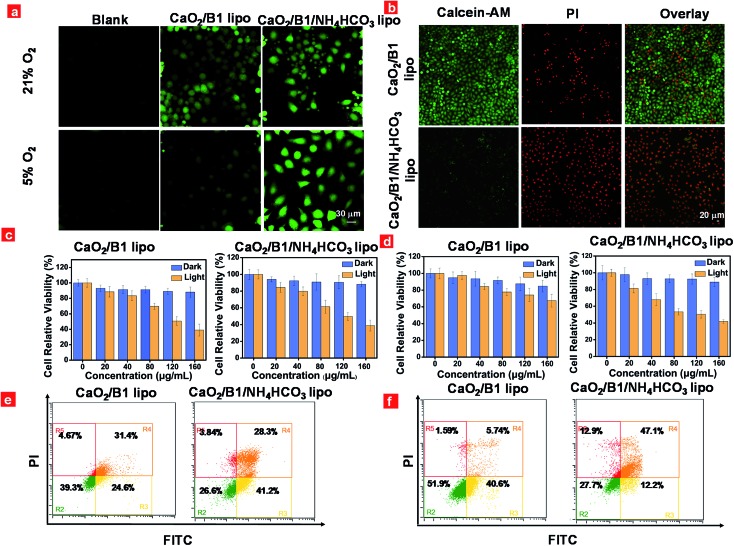
(a) ROS generation in cells incubated with CaO_2_/**B1** lipo and CaO_2_/**B1**/NH_4_HCO_3_ lipo (80 μg mL^–1^) under 21% and 5% oxygen levels and then exposed to irradiation; (b) calcein-AM/PI staining of cells incubated with CaO_2_/**B1** lipo and CaO_2_/**B1**/NH_4_HCO_3_ lipo (80 μg mL^–1^) under a 5% oxygen level and then exposed to irradiation; MTT assays of cells incubated with CaO_2_/**B1** lipo and CaO_2_/**B1**/NH_4_HCO_3_ lipo (80 μg mL^–1^) under 21% (c) and 5% (d) oxygen levels and then exposed to irradiation; flow cytometry results of cells incubated with CaO_2_/**B1** lipo and CaO_2_/**B1**/NH_4_HCO_3_ lipo (80 μg mL^–1^) under 21% (e) and 5% (f) oxygen levels and then treated with Annexin V-FITC/PI under irradiation. The excitation wavelength of irradiation is 730 nm. The power density is 500 mW cm^–2^. The irradiation time is 5 min.

The capacity of the liposomes to overcome the hypoxia-associated resistance towards PDT was examined *in vitro* using HeLa cells as a model through confocal laser-scanning luminescence microscopy. Light-induced intracellular generation of reactive oxygen species was assessed using 2,7-dichlorofluorescein diacetate (DCFH-DA) as an indicator. As illustrated in Fig. 4a, intracellular green fluorescence was observed in CaO_2_/**B1** lipo and CaO_2_/**B1**/NH_4_HCO_3_ lipo treated cells under irradiation for 5 min in air owing to the presence of photosensitizer **B1** units in the liposomes. However in the hypoxia environment, relatively intense fluorescence was detected in CaO_2_/**B1**/NH_4_HCO_3_ lipo treated cells compared to CaO_2_/**B1** lipo treated cells, which demonstrated that the good ability of the light-triggered oxygen production would be beneficial to ^1^O_2_ generation from **B1** in the intracellular hypoxia environment. As a control, blank cells or cells incubated with NH_4_HCO_3_ lipo and CaO_2_/NH_4_HCO_3_ lipo showed weak fluorescence under light stimulation in air and in the hypoxia environment (Fig. S8†).

Furthermore, the anticancer efficiency was investigated through calcine AM and propidium iodide (PI) co-staining assay. Different from the control groups containing blank cells or cells incubated with NH_4_HCO_3_ lipo and CaO_2_/NH_4_HCO_3_ lipo, cells treated with CaO_2_/**B1** lipo and CaO_2_/**B1**/NH_4_HCO_3_ lipo were totally damaged and displayed an intense red fluorescence signal from PI, while the green fluorescence from calcine AM was hardly observed in air (Fig. S9†). Notably, in the hypoxia environment, the damage effects of the cells incubated with CaO_2_/**B1** lipo were limited owing to insufficient oxygen supplementation (Fig. 4b). The cells incubated with CaO_2_/**B1**/NH_4_HCO_3_ lipo showed good anticancer efficiency after irradiation in the hypoxia environment. To determine whether the anticancer efficiency was attributed to the photothermal effect from **B1** units, cells preincubated with NAC, a ROS scavenger, and CaO_2_/**B1**/NH_4_HCO_3_ lipo were investigated after irradiation in air and the hypoxia environment (Fig. S11†). Only a green fluorescence signal was detected in the cells, revealing that the PDT effect was dominated to induce the cell death.

Consequently, the phototoxicity of the liposomes towards HeLa cells was assessed through the methyl thiazalyltetrazalium (MTT) assay. As illustrated in Fig. 4c and d, liposomes with different concentrations were added to the medium of cells. When the cells were incubated in the dark for 24 h, the liposomes treated cells sustained high cell viability (>80%) when the dose was less than 160 μg mL^–1^, demonstrating the low cytotoxicity of these liposomes towards HeLa cells. After irradiation, about 38.9% and 38.8% cell viability were observed when the cells were treated with CaO_2_/**B1** lipo and CaO_2_/**B1**/NH_4_HCO_3_ lipo, respectively at a concentration of 160 μg mL^–1^ in air (Fig. 4c), whereas in the hypoxia environment the values changed to 67.5% and 41.9% at the same concentration, respectively (Fig. 4d). The control groups, including cells incubated with NH_4_HCO_3_ lipo and CaO_2_/NH_4_HCO_3_ lipo, maintained good cell viability (>73%) in the absence and presence of irradiation (Fig. S12†). On the other hand, flow cytometry experiments by FITC and PI co-staining were conducted. In agreement with the above MTT assay, the percentage of late apoptotic cells treated with CaO_2_/**B1**/NH_4_HCO_3_ lipo reached 28.3% and 47.1% in air and the hypoxia environment, respectively (Fig. 4e and f), suggesting the good anticancer capacity of the oxygen self-sufficient CaO_2_/**B1**/NH_4_HCO_3_ lipo. Compared to other control groups, better anticancer capacity of CaO_2_/**B1**/NH_4_HCO_3_ lipo was observed in the hypoxia environment (Fig. 4f, S13 and S14†). All the results demonstrated that the light-amplified oxygen supplementation in CaO_2_/**B1**/NH_4_HCO_3_ lipo would be favorable to overcome the hypoxia-associated restriction towards PDT.

**Fig. 5:**
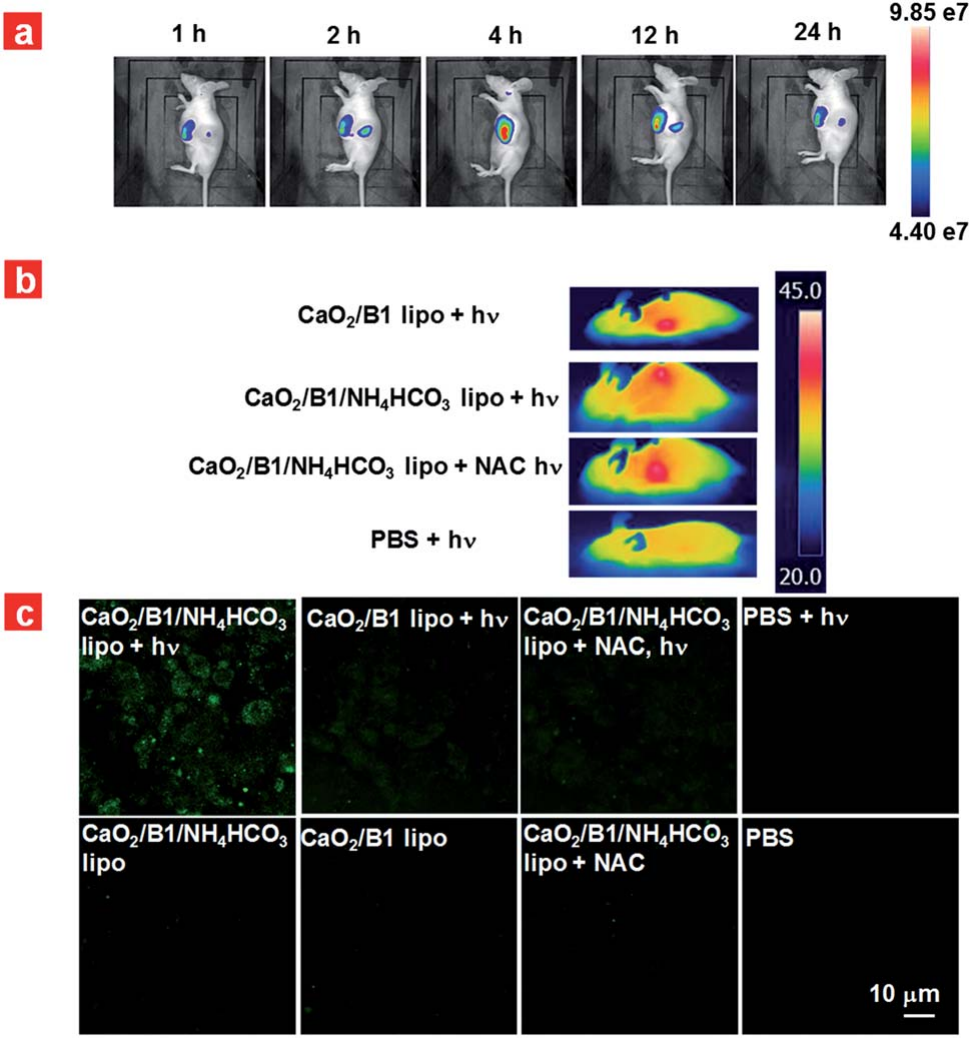
(a) Fluorescence images of a mouse bearing tumors monitored after tail intravenous injection of CaO_2_/**B1**/NH_4_HCO_3_ lipo (800 μg mL^–1^) at different times; (b) photothermal images of the mice bearing tumors with different treatments under irradiation (730 nm, 100 mW cm^–2^); (c) ROS generation of a tumor slice obtained from the mice with different treatments. “+*hν*” represents that the slice was from the mice exposed to irradiation (730 nm, 100 mW cm^–2^). NAC represents that it was pre-injected into the mice to scavenge ROS.

Encouraged by the intracellular results, we further verified the *in vivo* PDT effects of liposomes and examined the intratumoral anti-hypoxia capacity. HeLa tumor-bearing nude mice were employed as the model. CaO_2_/**B1**/NH_4_HCO_3_ lipo were intravenously injected into the mice and the biodistribution of the liposomes was tracked at 1 h, 2 h, 4 h, 12 h, and 24 h post-injection. Enhanced fluorescence in the tumor was exhibited at 1 h post-injection (Fig. 5a), revealing the significant accumulation of CaO_2_/**B1**/NH_4_HCO_3_ lipo there, which can be attributed to the enhanced permeability and retention effects. The fluorescence intensity reached a plateau at 4 h post-injection, and the tumorous fluorescence could be detected at 24 h post-injection. The fluorescence gradually decreased with the time prolonging owing to the metabolism of CaO_2_/**B1**/NH_4_HCO_3_ lipo (Fig. S15†). Compared to other major organs, the tumor exhibited the maximized biodistribution, supporting the fact that CaO_2_/**B1**/NH_4_HCO_3_ lipo exhibited good accumulation in the tumor (Fig. S16†).

Prior to the evaluation of anticancer efficiency *in vivo*, we first analysed the photothermal effects using 4 groups of tumor-bearing mice (Fig. 5b). After 4 h injection, the temperature of the tumor region was monitored under 730 nm laser irradiation (100 mW cm^–2^). No matter whether NAC was injected into the mice, mice treated with CaO_2_/**B1**/NH_4_HCO_3_ lipo and CaO_2_/**B1** lipo showed that the temperature of the tumor region reached up to about 45 °C (Fig. 5b and S17†). In the control group of PBS-treated mice, little temperature enhancement (0.46 °C) was observed. According to the temperature mapping results, the temperature of the tumor-surrounding region was not obviously affected by the irradiation. These results revealed that the good photothermal effects of liposomes containing **B1** played a key role in increasing the temperature in the tumor region and had no obvious damage to the surrounding tissues. To mainly investigate the photodynamic performance *in vivo*, we further exposed the mice under irradiation (730 nm, 100 mW cm^–2^) for 5 min for therapy to avoid the overheating of the tumor. The ROS generation of tumor slices was investigated using DCHF-DA as the indicator (Fig. 5c). The confocal image of slices from CaO_2_/**B1**/NH_4_HCO_3_ lipo injected mice exhibited a relatively more intense fluorescence signal compared to that from CaO_2_/**B1** lipo injected mice, indicating more ROS generation in the group of CaO_2_/**B1**/NH_4_HCO_3_ lipo. The phenomenon could be ascribed to the light-triggered photodynamic efficiency because the groups without irradiation or with NAC treatment displayed weak fluorescence.

**Fig. 6:**
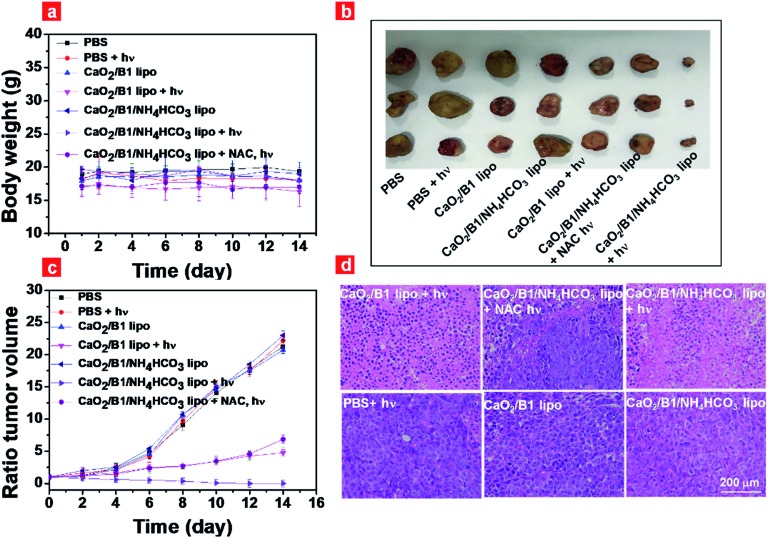
(a) Body weight of mice with different treatments during the therapy process; (b) photograph of tumors obtained from the mice after 14 days; (c) tumor volume ratio of mice that underwent different treatments during the therapy process; (d) H&E staining of tumor slices obtained from the different groups of mice after 14 days. “+*hν*” represents that the slice was from the mice exposed to irradiation (730 nm, 100 mW cm^–2^). NAC represents that it was pre-injected into the mice to scavenge ROS.

Inspired by the results of photothermal effects and ROS generation *in vivo*, we further estimated the regression degree of tumor growth to evaluate the anticancer efficiency of CaO_2_/**B1**/NH_4_HCO_3_ lipo. Mice that bear tumors were intravenously injected with different samples, followed by irradiation for 5 min or not. The body weight and tumor volume were monitored every 2 days. The body weight of the mice sustained between 16 and 19 g (Fig. 6a), and no abnormal behaviors were observed during therapy. Without irradiation, all groups showed rapid and similar enhancement of tumor volume and about a 20-fold increase was detected after 14 day treatment (Fig. 6b and c), suggesting the negligible effects of samples on tumor growth. When tumors were subjected to irradiation, the group of CaO_2_/**B1**/NH_4_HCO_3_ lipo inhibited tumor growth and induced tumor disappearance after 14 days although an obvious scar was found on the mice treated with CaO_2_/**B1**/NH_4_HCO_3_ lipo after 4 days, while the tumor volumes of the group treated with CaO_2_/**B1** lipo were observed to increase 4.7 fold. These results revealed that enough oxygen generation from CaO_2_/**B1**/NH_4_HCO_3_ lipo was favorable to produce ^1^O_2_ in the presence of photosensitizer **B1**, thus resulting in an irreversible oxidative injury in tumors. When CaO_2_/**B1**/NH_4_HCO_3_ lipo-treated mice were injected with NAC ahead of irradiation, the tumor growth remained, demonstrating limited photothermal effects on tumor growth owing to the low power density of laser irradiation. The associated photographs of mice treated with different samples and tumors are illustrated in Fig. S18.†

To corroborate the therapeutic efficiency of liposomes, we collected the slices of tumors and major organs at the end of treatment, which were investigated by hematoxylin & eosin (H&E) staining (Fig. 6d). With irradiation, cell damage and inflammatory lesion were found in the tumors of CaO_2_/**B1**/NH_4_HCO_3_ lipo-treated mice. The group treated with NAC also displayed tumor necrosis to some degree due to photothermal effects. However the morphology of tumor slices from the groups without irradiation was not affected, demonstrating the importance of irradiation for anticancer therapy. Major organs sliced after treatment displayed negligible pathological changes (Fig. S19†). Taken all the results *in vivo* together, oxygen self-sufficient CaO_2_/**B1**/NH_4_HCO_3_ lipo induced the dominated oxidative damage towards tumors under irradiation. What's more, the resistance of the intratumoral hypoxia environment was examined through immunofluorescence staining of the hypoxia inducible factor (HIF-1α) and carbonic anhydrase IX (CA9) (Fig. S20†). The tumor sliced from CaO_2_/**B1**/NH_4_HCO_3_ lipo-treated mice exhibited lower expression of HIF-1α and CA9 compared to the group of CaO_2_/**B1** lipo, which indicated that the light-triggered CaO_2_/**B1**/NH_4_HCO_3_ lipo could relieve the intratumoral hypoxia environment.

## Conclusions

In summary, we have exhibited a proof-of-concept study that CaO_2_/**B1**/NH_4_HCO_3_ lipo can be employed as an oxygen self-sufficient nanomaterial to overcome hypoxia-associated restriction towards PDT. Under irradiation, the liposomes self-sufficiently triggered oxygen generation accompanied by clean by-products. Furthermore, the light-triggered oxygen supplementation favoured enhanced ^1^O_2_ generation in the presence of the photosensitizer (**B1**), thus resulting in improved anticancer efficiency. In the context of good therapy effects *in vitro*, we estimated the administration of intravenous injection of the liposomes in mice. The liposomes were accumulated in the tumor after 4 h injection. The relief of the hypoxia environment *in vivo* was observed through immunofluorescence staining of HIF-1α and CA9. The enhanced ^1^O_2_ generation induced an oxidative damage towards the tumor, and further led to a good inhibitory effect on the tumor growth. In this work, the utilization of the light-activatable oxygen self-sufficient CaO_2_/**B1**/NH_4_HCO_3_ lipo offers a valuable attempt to regulate intratumoral hypoxia and overcome the limitation of current PDT. To our knowledge, this highlights the first example of using NIR light to activate CaO_2_ nanoparticle-containing liposomes for the modulation of the hypoxic environment in tumors.

## Experimental section

The detailed information of materials, instruments, synthesis and characterization of liposomes, cell culture, and *in vitro*/*in vivo* experiments can be found in the ESI.†

## Animal models

All the nude mice were purchased from the Comparative Medicine Center of Yangzhou University. All the animal experiments were conducted in line with the specifications of The National Regulation of China for Care and Use of Laboratory Animals and approved by the Jiangsu Administration of Experimental Animals.

## Conflicts of interest

There are no conflicts to declare.

## Supplementary Material

Supplementary information
