# Supplementary material for: Oxygen self-sufficient NIR-activatable liposomes for tumor hypoxia regulation and photodynamic therapy
Source: Chem Sci. 2019 Aug 8;10(39):9091–8. doi: 10.1039/c9sc03161h (PMC6889832; doi:10.1039/c9sc03161h)
Supplement: Supplementary file 1 [file SC-010-C9SC03161H-s001.pdf]

Electronic Supplementary Material (ESI) for Chemical Science.
This journal is © The Royal Society of Chemistry 2019

Electronic Supplementary Information (ESI)

Oxygen self-sufficient NIR-activatable liposomes for tumor hypoxia regulation and photodynamic therapy

Qi Yu,[‡a] Tianci Huang,[‡a] Chao Liu,[a] Menglong Zhao,[a] Mingjuan Xie,[a] Guo Li,[a] Shujuan Liu,[a] Wei Huang*[a,b] and Qiang Zhao*[a]

a. Key Laboratory for Organic Electronics and Information Displays & Jiangsu Key Laboratory for Biosensors, Institute of Advanced Materials (IAM), Nanjing University of Posts and Telecommunications (NUPT), Nanjing 210023, P. R. China.

b. Shaanxi Institute of Flexible Electronics (SIFE), Northwestern Polytechnical University (NPU), Xi'an 710072, Shaanxi, P. R. China.

Email: iamqzhao@njupt.edu.cn; provost@nwpu.edu.cn.

1. Materials

NH_4HCO_3, DPPC and cholesterol were purchased from Sigma-Aldrich. DSPE-PEG5000 were purchased from Xi'an Ruixi Biological Technology Co., Ltd. $CaCl_2$ and H_2O_2 (30 w.t. % in water) were purchased from Aladdin. Cell culture reagents and fetal bovine serum (FBS) were supplied from Beyotime Biotechnology. All reagents were purchased from commercial suppliers and used without further purification.

2. Instruments

NMR spectra were recorded on a Bruker Ultra Shield Plus 400 MHz NMR instrument (^1H: 400 MHz, ^{13}C:100 MHz). Chemical shifts (ppm) were reported relative to tetramethylsilane (TMS). Mass spectra were obtained on a Bruker autoflex matrix-assisted laser desorption/ionization time of flight mass spectrometer (MALDI-TOFMS). TEM images were obtained from Hitachi TEM system. The average hydrodynamic size was recorded on ZetaPALS analyser (Brookhaven, USA). XRD analysis were performed via a Bruker D8 Advanced. UV-Vis absorption and photoluminescence spectra were measured via a Shimadzu UV-3600 UV-VIS-NIR spectrophotometer and Edinburgh FL 920 spectrophotometer, respectively. The photothermal effects were recorded using a thermal infrared imager (FLIR E40). The concentration of oxygen generated in aqueous solution were quantified by a dissolved oxygen meter (HY30D53301000, HACH, USA). Confocal luminescence images carried out by a laser scanning confocal microscopy (Olympus Fluo view FV1000) equipped with 20× objective lens. Photographs of the mice were taken with a Cannon EOC 400D digital camera.

3. Synthesis and characterization of B1

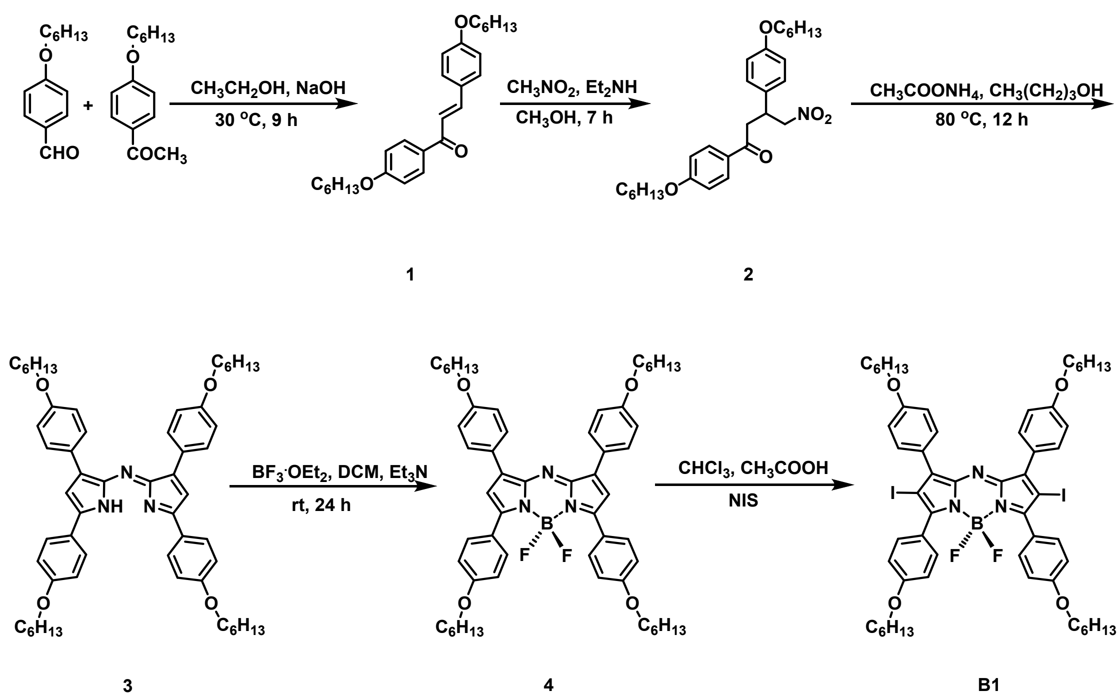

Compound 1-4 and **B1** were synthesized according to the previous work.[1]

Compound 1: ^{1}H NMR (400MHz, $CDCl_3$) δ (ppm) : 8.02 (d, J = 7.2 Hz, 2 H), 7.77 (d, J = 15.6 Hz, 1 H), 7.58 (d, J = 8.4 Hz, 2 H), 7.43 (d, J = 15.6 Hz, 1 H), 6.96 – 6.91 (m, 4 H), 4.08 – 3.97 (m, 4 H), 1.88 – 1.74 (m, 4 H), 1.49 – 1.25 (m, 12 H), 0.91 (t, J = 13.6 Hz, 6 H).

Compound 3: ^{1}H NMR (400MHz, $CDCl_3$) δ (ppm) : 8.02 (d, J = 8.8 Hz, 4 H), 7.84 (d, J = 8.8 Hz, 4 H), 7.01 (d, J = 8.3 Hz, 4 H), 6.99 (s, 2 H), 6.94 (d, J = 12.8 Hz, 4 H), 4.07 – 3.99 (m, 8 H), 1.88 – 1.77 (m, 8 H), 1.55 – 1.45 (m, 8 H), 1.42 – 1.33 (m, 16 H), 0.97 – 0.89 (t, J = 6.8 Hz, 12 H).

Compound 4: ^{1}H NMR (400MHz, $CDCl_3$) δ (ppm) : 8.08 (d, J = 2.0 Hz, 4 H), 8.06 (d, J = 2.0 Hz, 4 H), 7.02 (d, J = 2.2 Hz, 4 H), 6.99 (d, J = 2.2 Hz, 4 H), 6.95 (s, 2 H), 4.08 – 4.03 (m, 8 H), 1.88 – 1.79 (m, 8 H), 1.45 – 1.32 (m, 24 H), 0.98 – 0.96 (m, 12 H).

B1: ^{1}H NMR (400MHz, $CDCl_3$) δ (ppm) : 7.83 (d, J = 8.8 Hz, 4 H), 7.67 (d, J = 8.9 Hz, 4 H), 7.01 – 6.95 (m, 8 H), 4.07 – 3.98 (m, 8 H), 1.88 - 1.78 (m, 8 H), 1.55 – 1.43 (m, 8 H), 1.42 – 1.35 (m, 16 H), 0.98 – 0.91 (m, 12 H).

^{13}C NMR (100 MHz, $CDCl_3$) δ (ppm): 161.08, 160.39, 160.11, 147.25, 144.80, 132.38, 132.33, 124.44, 123.29, 113.94, 113.79, 68.13, 67.99, 31.62, 31.58, 29.23, 29.21,

25.78, 25.76, 22.65, 22.63, 14.07, 14.06.

4. Synthesis of CaO_2 nanoparticles

CaO_2 nanoparticles were prepared according to the previous work reported by Khodaveisi *et al.*[2]. $CaCl_2$ (3 g) were dissolved in 30 mL ultrapure water, followed by adding 15 mL NH_3•H_2O (1 M) and 94 mL PEG200. Then 15 mL H_2O_2 (30%) was gradually added to the mixture at the rate of three drops per min. The mixture was further stirred for another 1 h. To precipitate the prepared nanoparticles, pH value of the mixture was adjusted to about 11 using NaOH (0.1 M). Finally, CaO_2 nanoparticles were obtained by centrifugation at 10000 rpm for 5 min and washed three times with NaOH solution and ultrapure water.

5. Synthesis of liposomes

DPPC (5 mg), cholesterol (1.8 mg) and DSPE-PEG5000 (3.2 mg) were mixed with **B1** (1 mg) and dissolved in chloroform. Then 200 μL CaO_2 nanoparticles (10 mg/mL) dispersed in ethanol were added. The mixture was dried via a rotary evaporator under a decreased pressure to form a lipid film. 10 mL NH_4HCO_3 (2.7 M) were added into the lipid film, following the continuous sonication (360 W) at room temperature for 30 s. The suspension was centrifuged at 10000 rpm for 10 min, and washed with three times with ultrapure water to remove the unencapsulated NH_4HCO_3. The control groups of liposomes were prepared according to a similar procedure without adding **B1**, CaO_2 nanoparticles or NH_4HCO_3. Finally, the liposomes were dispersed in PBS buffer.

6. Evaluation of $^1O_2$ generation of liposomes

1,3-diphenylisobenzofuran (DPBF) was used as a $^1O_2$ indicator. The air-saturated liposomes (184 μg/mL) dispersed in PBS buffer containing 10 μL DPBF (500 μM) was prepared. The solution in the hypoxia environment was produced by bubbling with Argon. The solution was irradiated under a NIR 730 nm laser (500 mW/cm^2) in an

interval of 1 min at 37 °C.

7. Cell culture

HeLa cell lines were provided by the Institute of Biochemistry and Cell Biology, SIBS, CAS (China). The cells were grown in DMEM (Dulbecco's modified Eagle's medium) supplemented with 10% FBS (fetal bovine serum), 100 mg/mL streptomycin and 100 U/mL penicillin at 37 °C with 5% CO_2.

8. Cellular uptake of liposomes

HeLa cells were incubated in DMEM (high glucose) medium containing 10% FBS at 37 °C in a humidified atmosphere containing 5% CO_2. HeLa cells were seeded in the confocal dishes at a density of 10^5 cells/dish. After 24 h incubation, cells were attached and treated with free medium containing liposomes (80 μg/mL) for another 2 h.

9. Cellular ROS generation

Cells were cultured with different groups of liposomes in normoxic or hypoxic condition for 2 h, followed by the treatment of the commercial ROS indicator, DCFH-DA (10 μM) for another 2 h. Then cells were irradiated by the laser (730 nm 500 mW/cm^2) for 5 min. The fluorescence from DCF was collected (λ_{ex} = 488 nm, λ_{em} = 500 – 540 nm). Cells were cultured with an atmosphere of 21% O_2 and 5% O_2 at 37 °C to mimic the normoxic and hypoxic environment, respectively.

10. Calcein-AM /PI assays

The viability of cells incubated with liposomes was evaluated via Calcein-AM/propidium iodide (PI) staining. Cells were cultured at 100% humidity 37 °C with 5% CO_2 for 24 h. Liposomes (80 μg/mL) were added to the medium and cells were incubated for another 2 h under 5% or 21% O_2. Then the cells were treated with PI (5 μL) and Calcein-AM (10 μL) in dark for 10 min and then irradiated with a 730 nm laser (500 mW/cm^2) for 5 min. The images were obtained by the confocal microscopy in green channel for Calcein-AM (λ_{ex} = 488 nm, λ_{em} = 500 – 560 nm) and red channel for

PI (λ_{ex} = 488 nm, λ_{em} = 600 – 680 nm) after 1 h.

11. Cell viability

The cytotoxicity of liposomes towards HeLa cells was assessed by the methyl thiazolyl tetrazolium (MTT) assay. HeLa cells in log phase were seeded into a 96-well cell-culture plate at 1×10^4/well, followed by incubation at 37 °C and 5% CO_2 atmosphere for 24 h. The liposomes at concentrations of 20, 40, 80, 120, 160 μg/mL was added to the wells. To evaluate the dark cell viability, the cells were incubated in dark at 37 °C and 5% CO_2 atmosphere for 24 h. To assess the phototoxicity, cells were irradiated with a 730 nm laser (500 mW/cm^2) for 5 min and then further incubated for 24 h. 20 mL MTT solution (5 mg mL^{-1}) was added to each well of the 96-well assay plate, and the solution was incubated for another 3 h under the same condition. The OD570 (absorbance value) of each well referenced at 690 nm were measured using a Tecan Infinite M200 monochromator based multifunction microplate reader. The following formula was used to calculate the viability of cell growth: viability (%) = [(mean of absorbance value of treatment group)/(mean absorbance value of control)]×100%.

12. Annexin V-FTIC/PI assays

HeLa cells were seeded in the 6-well plates and incubated at 100% humidity 37 °C with 5% CO_2 for 24 h. Liposomes (80 μg/mL) were added to the medium and cells were incubated for another 2 h under 5% or 21% O_2. Then cells were irradiated with a 730 nm laser (500 mW/cm^2) for 5 min and further incubated in dark for 24 h under 5% or 21% O_2. Finally, cells were stained with Annexin-FITC/PI for 15 min and digested using trypsin and collected in centrifuge tubes. Cells were then analyzed by flow cytometry.

13. Animal model

The female athymic nude mice were purchased from Comparative Medicine Center of Yangzhou University (Permit number: 201825343) with relevant laws and guidelines. Mice bearing tumor models were established by subcutaneous injection of HeLa cells (about 10^6 per mouse) into the flanks of the mice. When the tumor volume reached

to about 100 mm^3, we began to conduct the relevant experiments.

14. *In vivo* therapy

Mice were divided into 7 groups and injected with **CaO_2/B1 lipo** (800 μg/mL, 100 μL), **CaO_2/B1/NH_4HCO_3 lipo** (800 μg/mL, 100 μL), or PBS. After 4 h, the groups treated with liposomes was injected with NAC (50 mM) or not and exposed under irradiation with a 730 nm laser (100 mW/cm^2) for 5 min or not. The tumor volume and body weight were estimated every 2 days for 14 days. The tumor volume was calculated according to the follow formula: $V = length \times width^2/2$. The ratio tumor volume V/V_0 was measured for investigation. After 14 days, the mice were sacrificed. Tumors and major organs were collected and fixed using 4% formalin solution for further H&E staining and immunofluorescence staining of CA9 and HIF-1α.

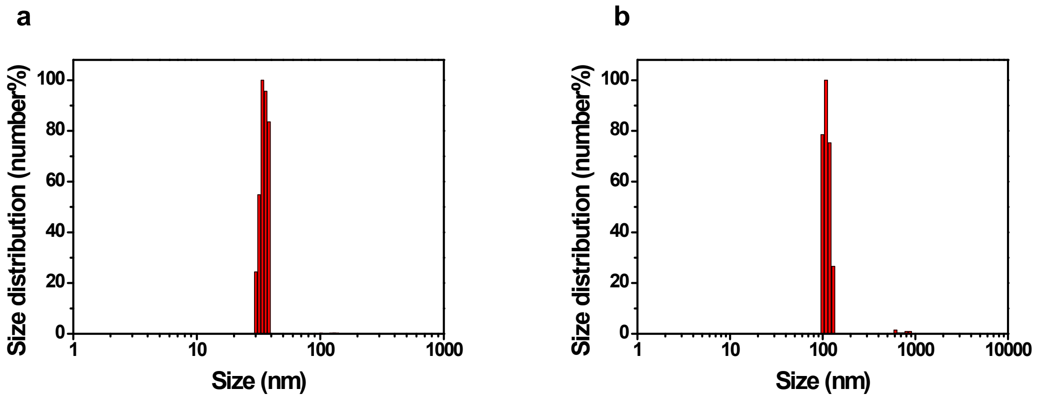

Fig. S1 The DLS results of CaO_2 nanoparticles and **CaO_2/B1/NH_4HCO_3 lipo**.

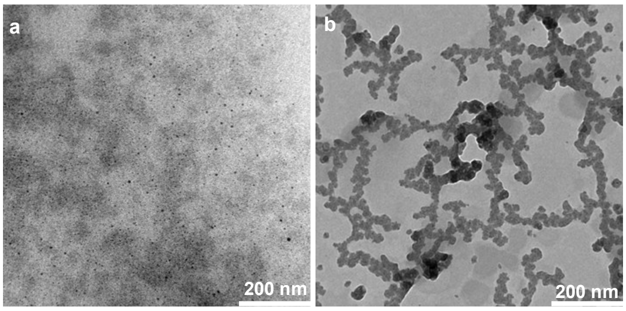

Fig. S2 TEM images of **CaO_2/B1/NH_4HCO_3 lipo** prepared under the sonication power of 600 W (a) and 180 W (b).

Since the size of liposomes is associated with sonication power, we also prepared **CaO_2/B1/NH_4HCO_3 lipo** under sonication power of 180 W and 600 W, and characterized the morphology of the liposomes. As seen in **Fig. S2**, when the power was 600 W, the lipid membrane and CaO_2 nanoparticles were observed in the TEM image, which may be ascribed to the instability of liposomes. When the power was decreased to 180 W, **CaO_2/B1/NH_4HCO_3 lipo** were aggregated and difficult to be well dispersed in PBS buffer. Thus we chose the liposomes prepared under sonication power of 360 W for further investigation.

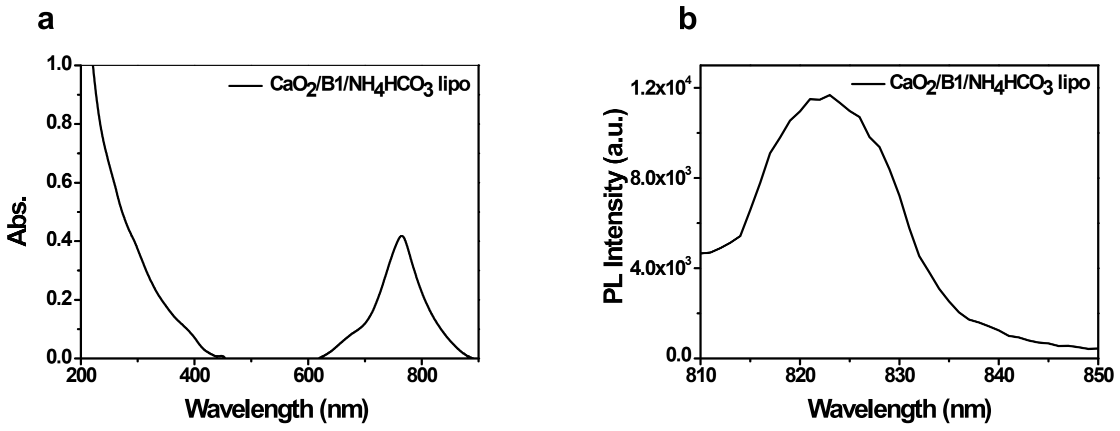

Fig. S3 The absorption and emission spectra of **CaO_2/B1/NH_4HCO_3 lipo**.

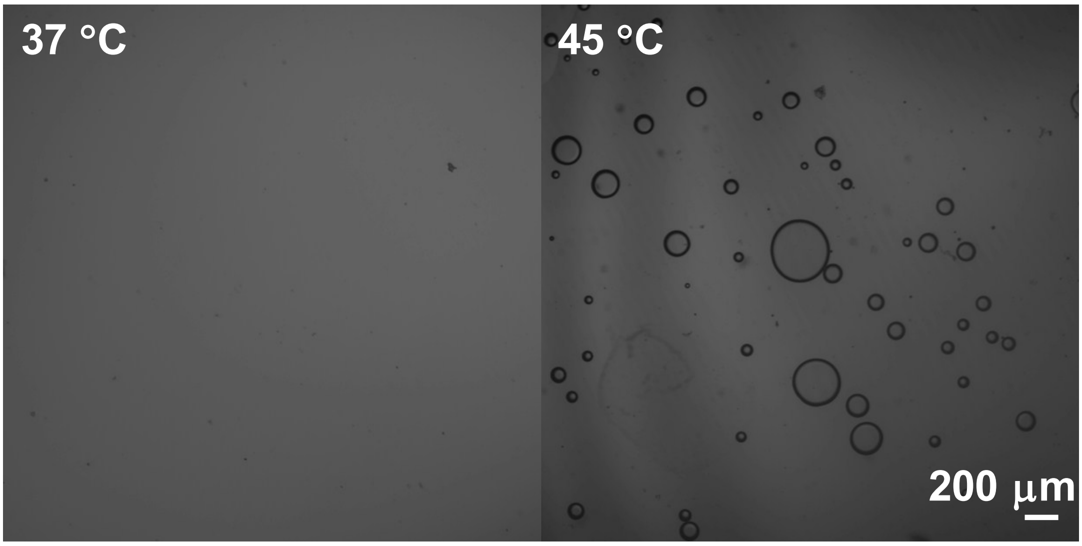

Fig. S4 Bright images of PBS buffer containing **NH_4HCO_3 lipo** at 37 and 45 °C.

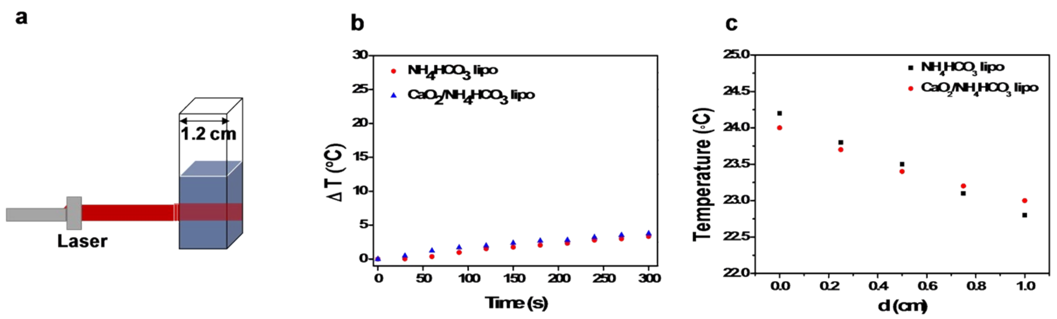

Fig. S5 (a) Schematic diagram of the evaluation of photothermal effects; (b) temperature changes of **NH_4HCO_3 lipo** or **CaO_2/NH_4HCO_3 lipo** (246 μg/mL) under different irradiation time; (c) temperature of **NH_4HCO_3 lipo** or **CaO_2/NH_4HCO_3 lipo** under different distance in the longitudinal axis away from the laser beam after irradiation for 5 min. The irradiation power is 192 mW. Beam area is 0.38 cm^2.

To investigate the heat diffusion, we tested the point temperature in the cell containing the liposomes after irradiation for 5 min (Fig. S5c). The d represents the distance in the longitudinal axis away from the laser beam. The temperature varied from ~24 to ~23 °C with the distance increased. The small changes of temperature could be ascribed to the absence of photothermal molecule **B1** in the liposomes.

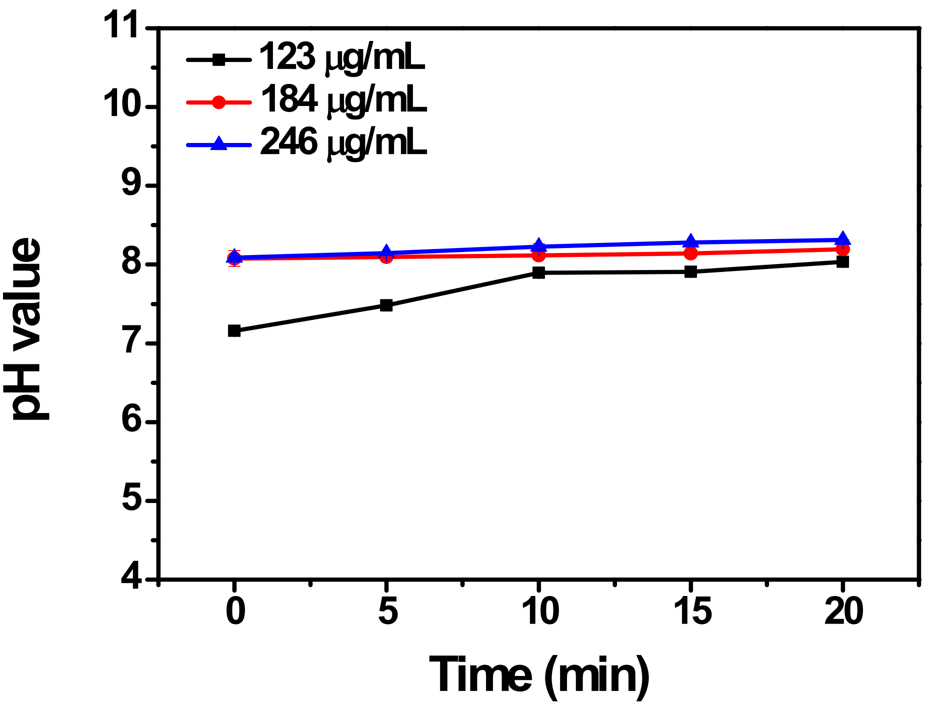

Fig. S6 pH values of **CaO_2/B1/NH_4HCO_3 lipo** with different concentrations under irradiation for 20 min.

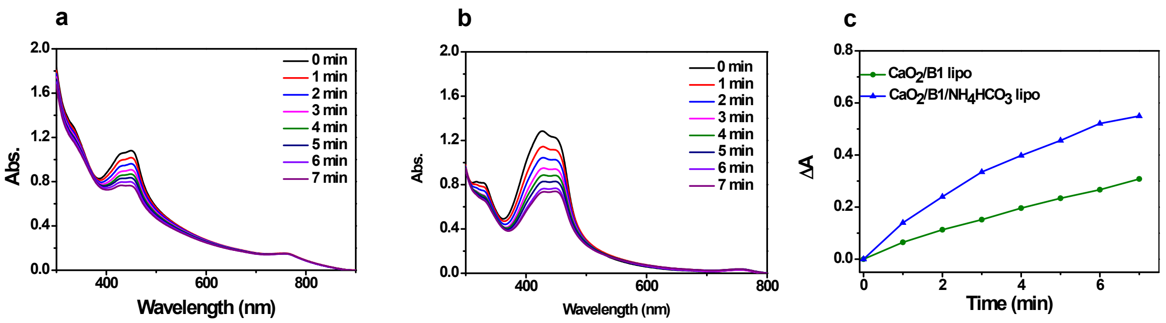

Fig. S7 Absorption spectra of the mixture containing DPBF and **CaO_2/B1 lipo** (a), **CaO_2/B1/NH_4HCO_3 lipo** (b) in air at different irradiation time in air at 37 °C; (c) ΔA of DPBF at 427 nm which are obtained from figure a and b. $\Delta A = A_t - A_0$, A_t was the absorbance of DPBF at 427 nm at different irradiation time and A_0 was the absorption without irradiation.

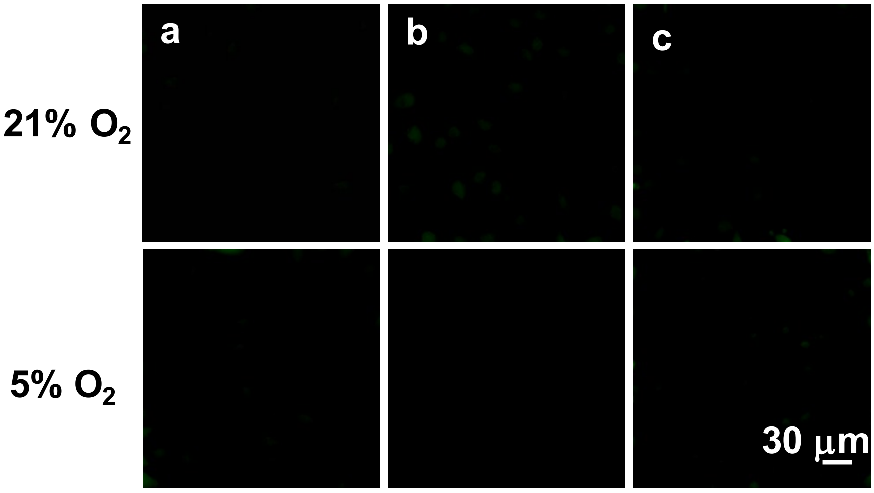

Fig. S8 ROS generation in blank cells (a), cells incubated with **NH_4HCO_3 lipo** (b) or **CaO_2/NH_4HCO_3 lipo** (c) (80 μg/mL) under 21% or 5% oxygen level and then exposed under 730 nm laser irradiation (500 mW/cm^2) for 5 min.

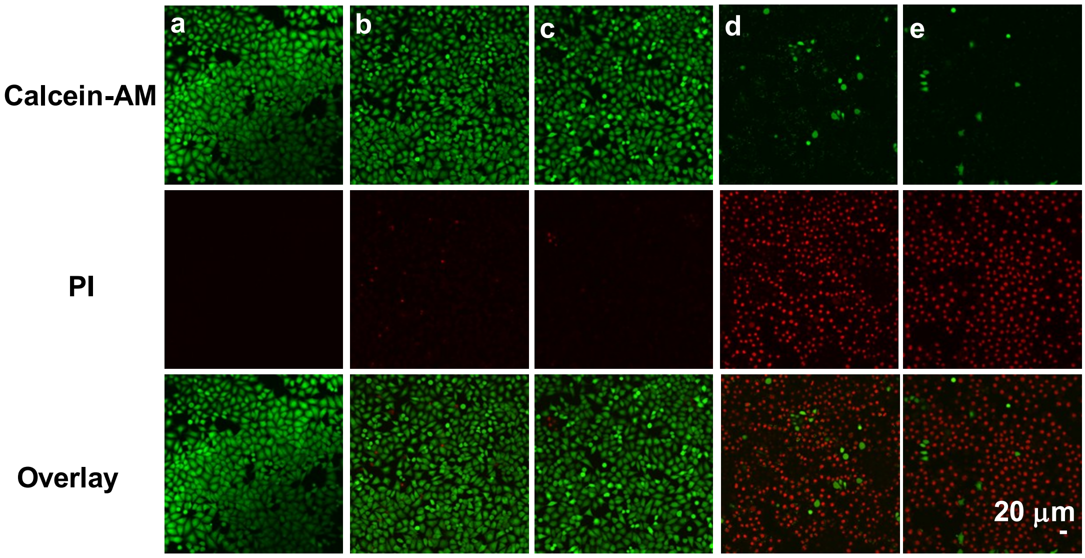

Fig. S9 Calcein-AM/PI assays of blank cells (a), cells incubated with **NH_4HCO_3 lipo** (b), **CaO_2/NH_4HCO_3 lipo** (c), **CaO_2/B1 lipo** (d), **CaO_2/B1/NH_4HCO_3 lipo** (e) (80 μg/mL) under 21% oxygen level and then exposed under 730 nm laser irradiation (500 mW/cm^2) for 5 min.

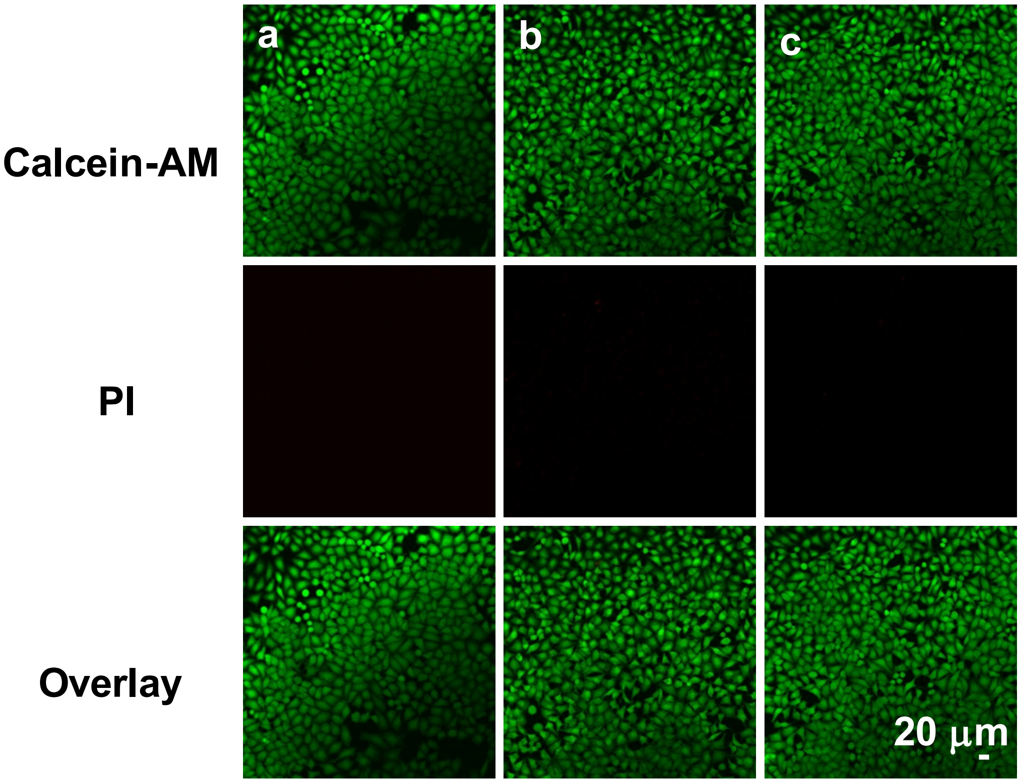

Fig. S10 Calcein-AM/PI assays of blank cells (a), cells incubated with **NH_4HCO_3 lipo** (b) or **CaO_2/NH_4HCO_3 lipo** (c) (80 μg/mL) under 5% oxygen level and then exposed under 730 nm laser irradiation (500 mW/cm^2) for 5 min.

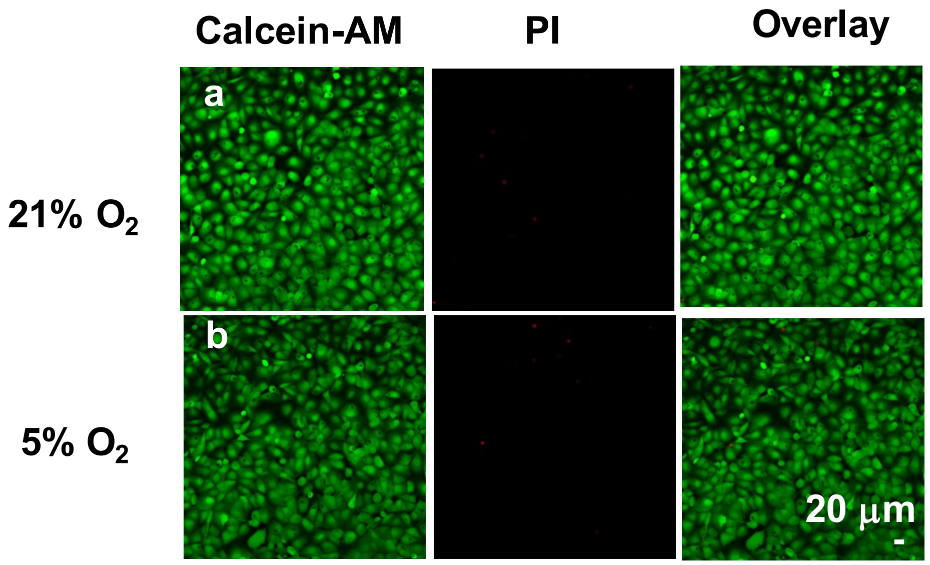

Fig. S11 Calcein-AM/PI assays of cells incubated with **CaO_2/B1/NH_4HCO_3 lipo** (80 μg/mL) and further treated with NAC (5 mM) under 730 nm laser irradiation (500 mW/cm^2) for 5 min under 21% (a) and 5% (b) oxygen level.

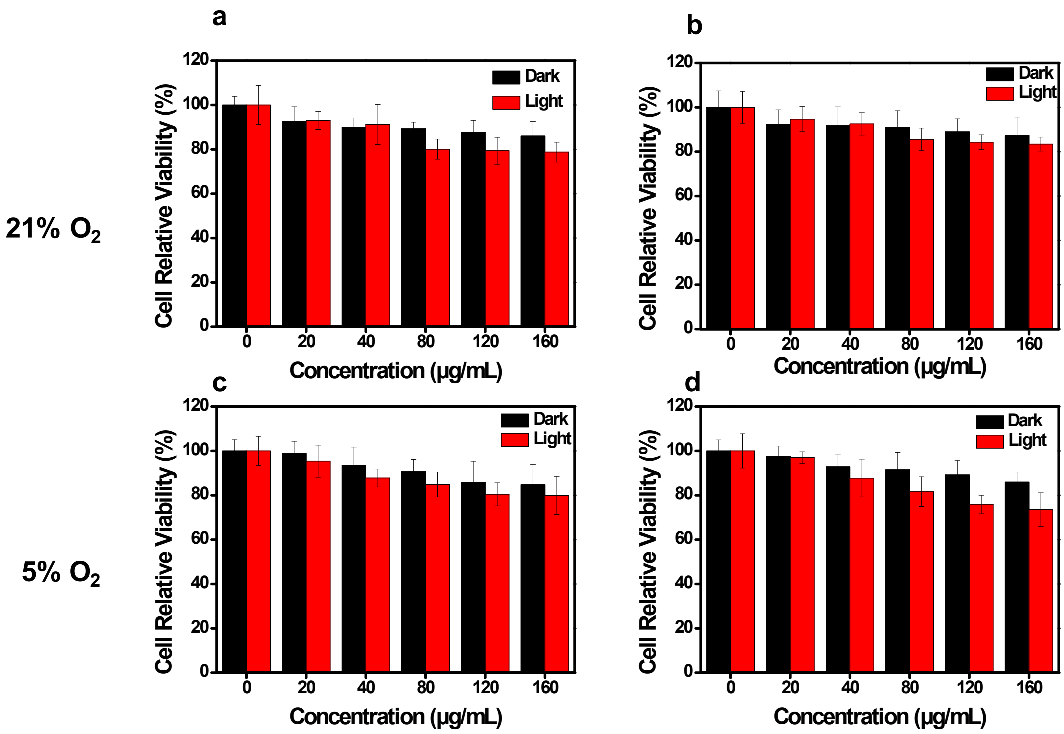

Fig. S12 MTT assays of cells incubated with (a, c) **NH_4HCO_3 lipo** or **CaO_2/NH_4HCO_3 lipo** (b, d) under 21% and 5% oxygen atmosphere with and without irradiation.

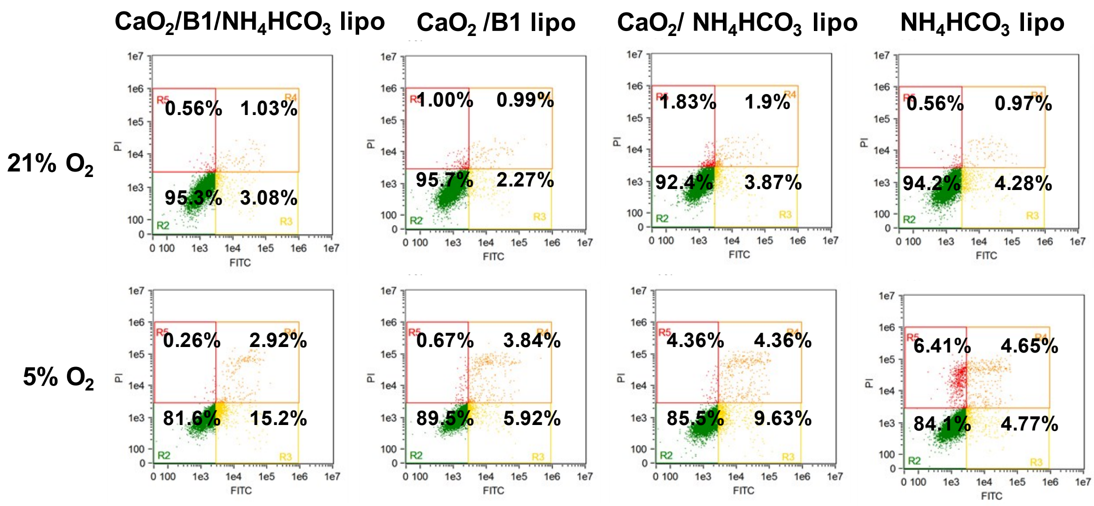

Fig. S13 Flow cytometry results of cells incubated with different liposomes (80 μg/mL) under 21% and 5% oxygen atmosphere without irradiation.

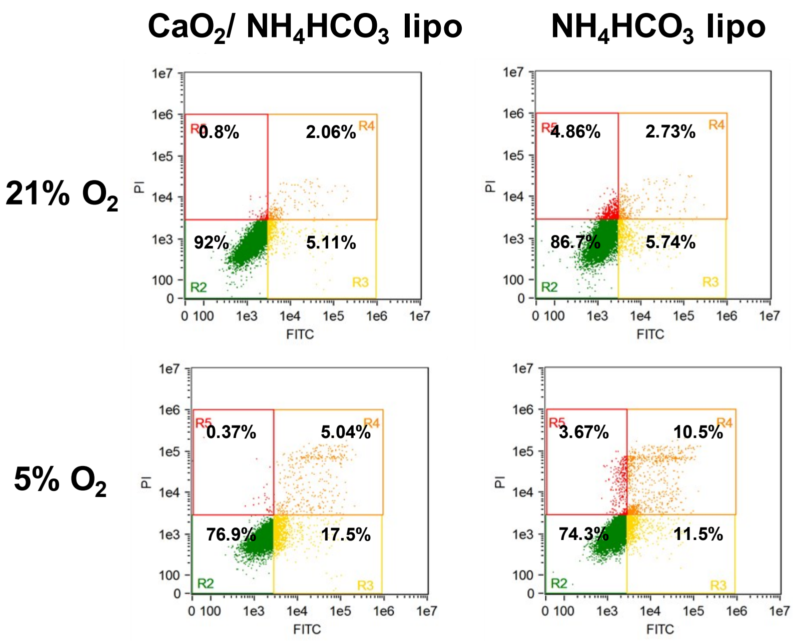

Fig. S14 Flow cytometry results of cells incubated with **CaO_2/NH_4HCO_3 lipo** or **NH_4HCO_3 lipo** (80 μg/mL) under 21% and 5% oxygen atmosphere with irradiation (730 nm 500 mW/cm^2).

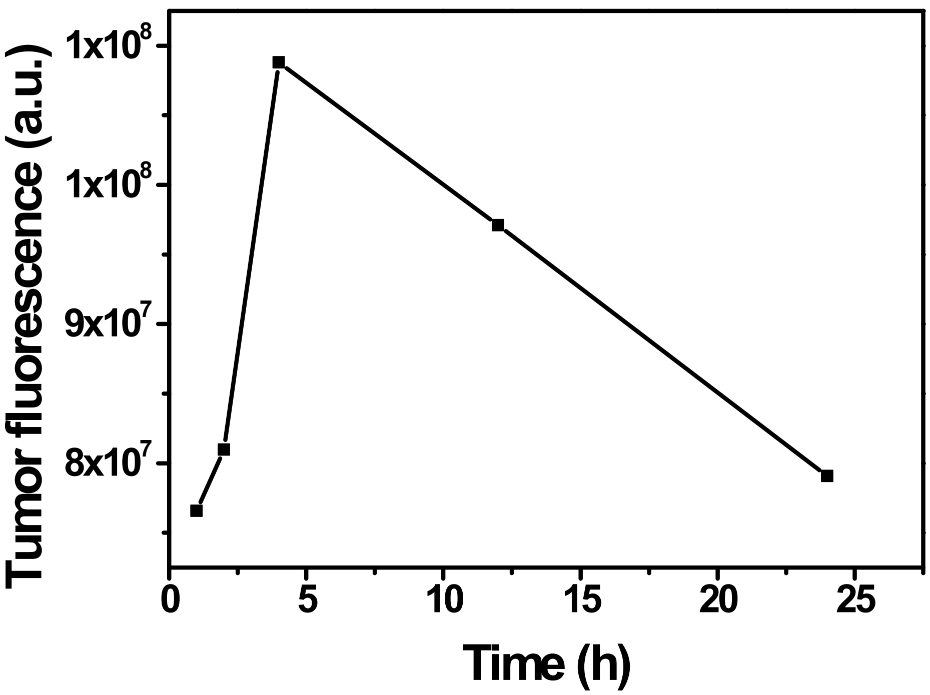

Fig. S15 The tumorous fluorescence monitored at different time.

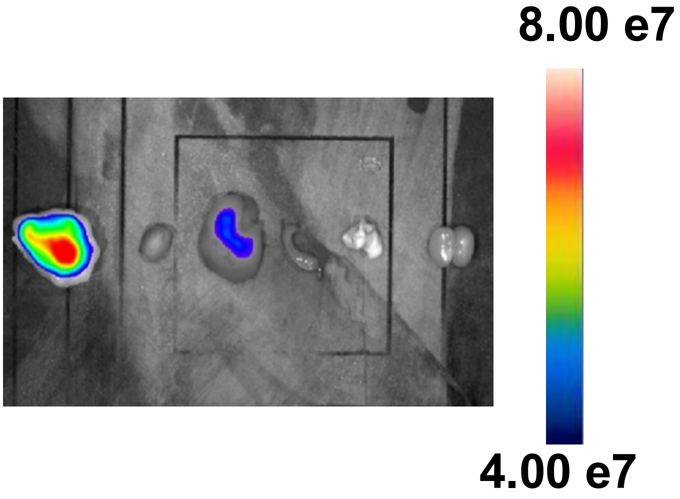

Fig. S16 Fluorescence images of the main organs of the mouse after 4 h injection of **CaO_2/B1/NH_4HCO_3 lipo**. From left to right is tumor, heart, liver, spleen, lung and kidney.

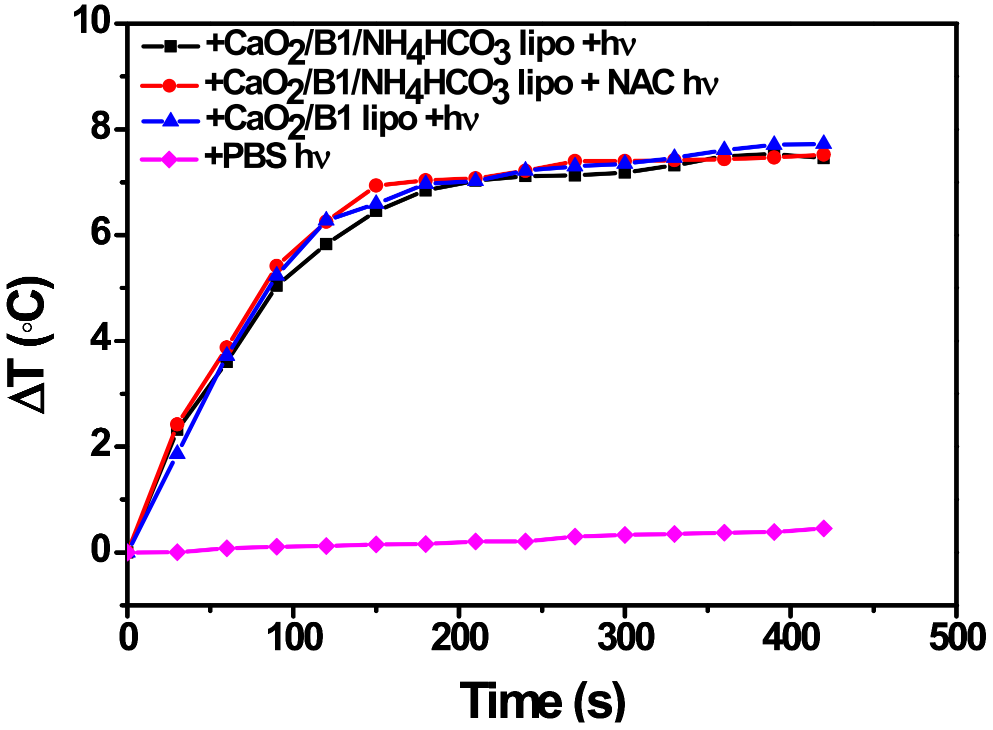

Fig. S17 Temperature changes of tumors with different treatments under laser irradiation for 7 min (730 nm, 100 mW/cm^2).

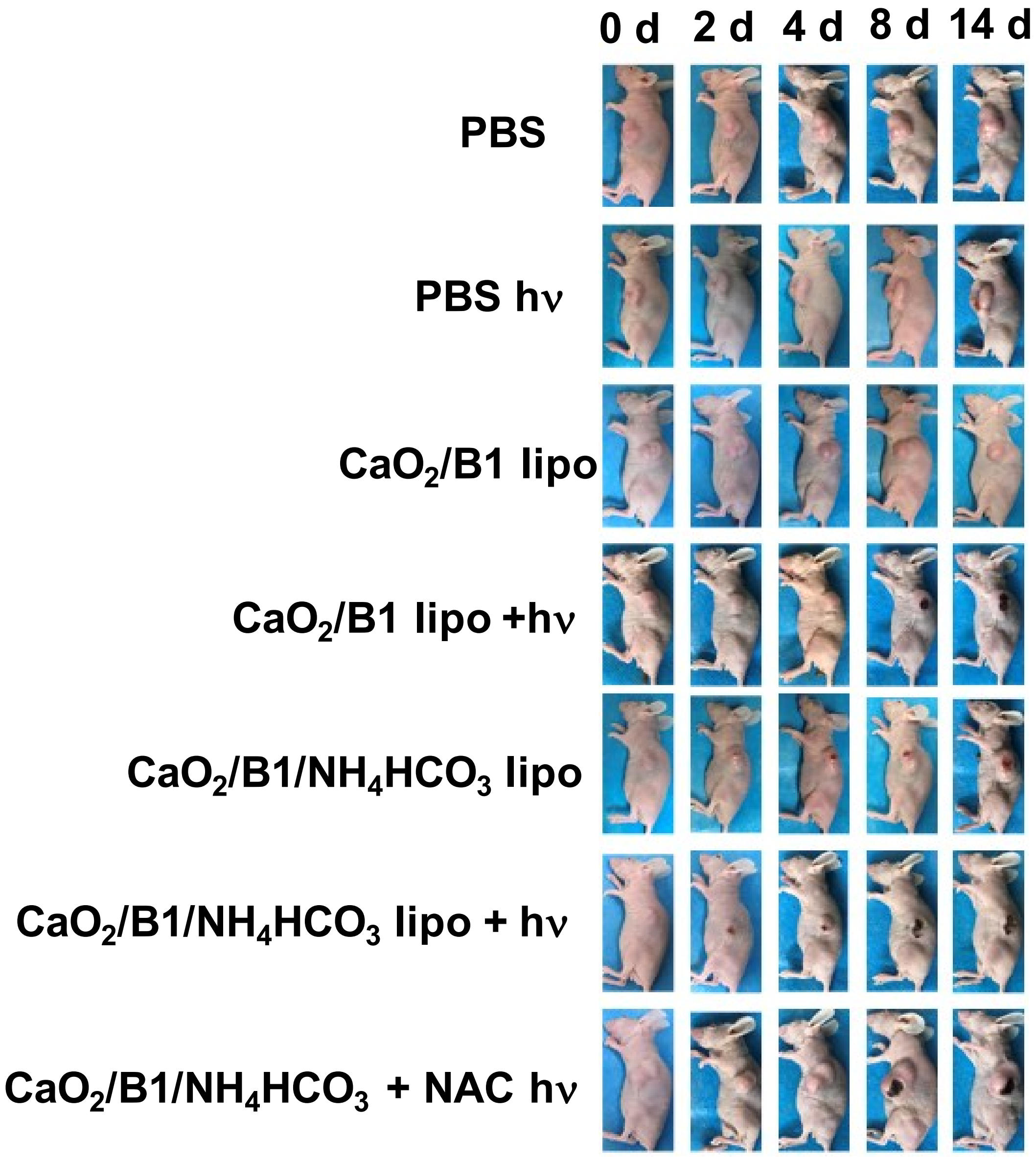

Fig. S18 Photographs of mice treated with different groups during therapy procedure.

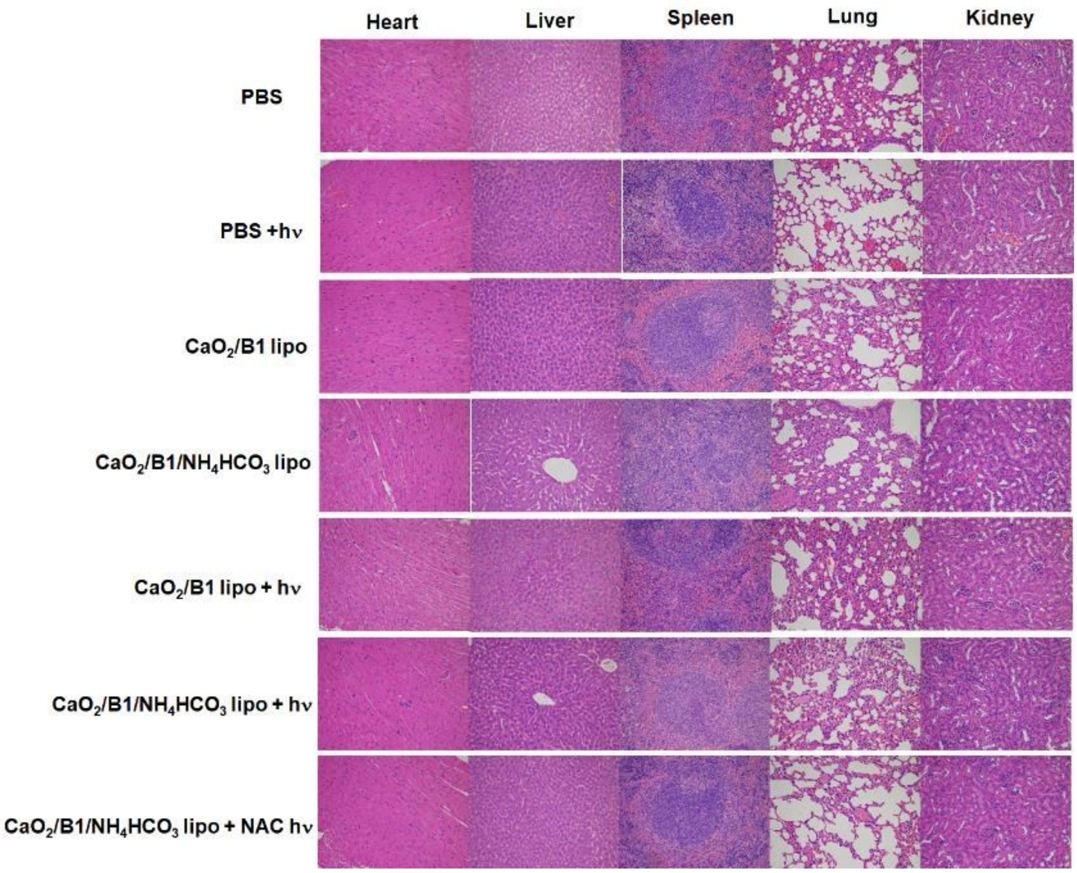

Fig. S19 The H&E stained slices harvested from the main organs of mice after different treatments.

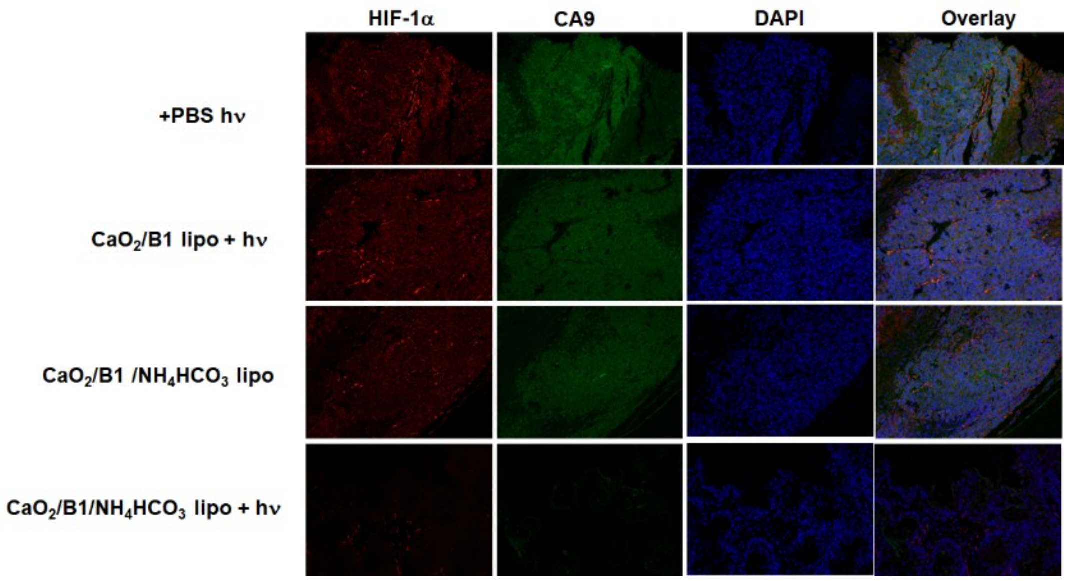

Fig. S20 Immunofluorescence staining of HIF-1α and CA9 of tumor slices with different treatments.

^{1}H NMR spectrum of compound 1

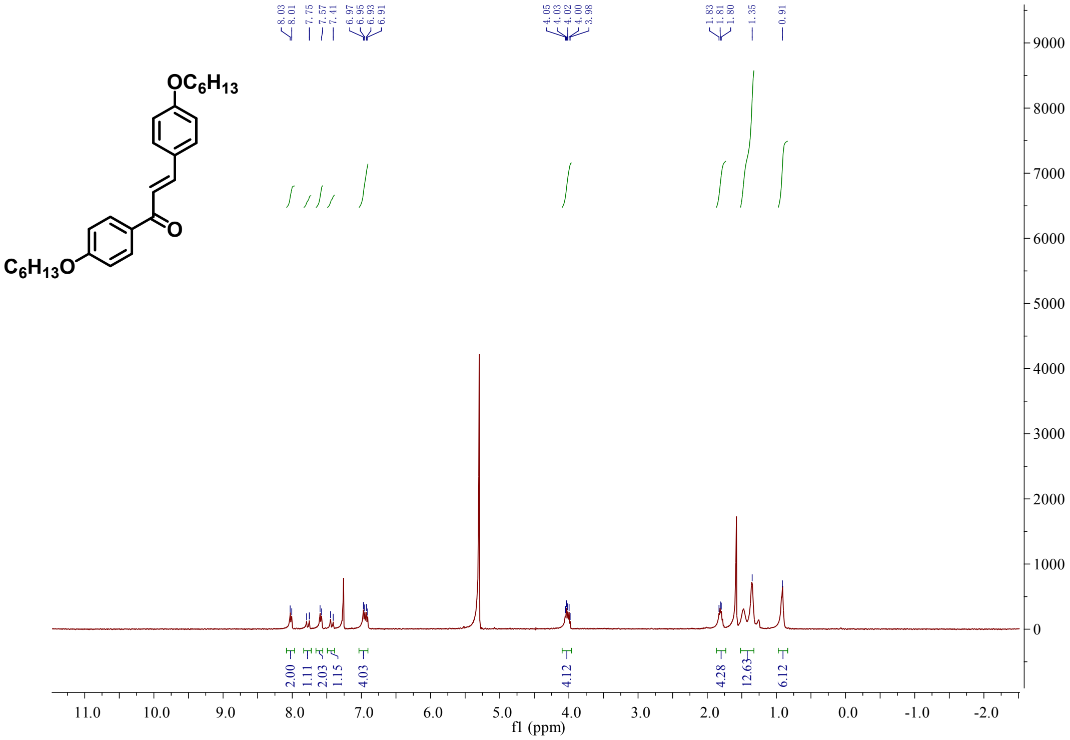

^{1}H NMR spectrum of compound 3

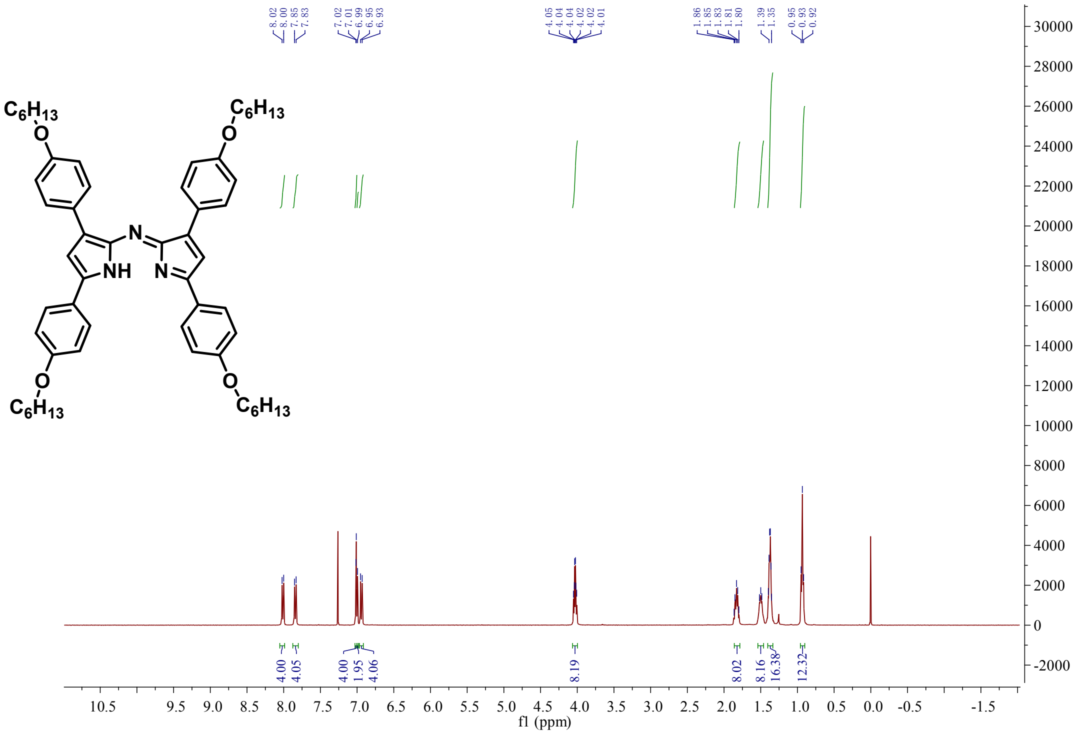

^{1}H NMR spectrum of compound 4

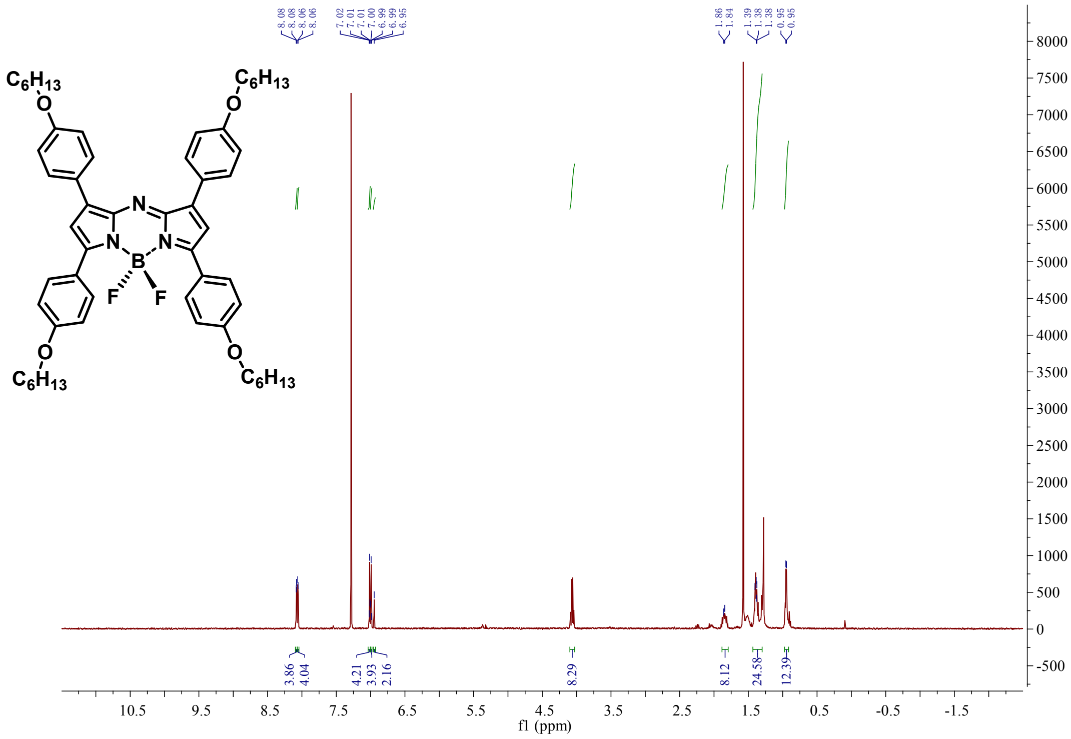

^{1}H NMR spectrum of B1

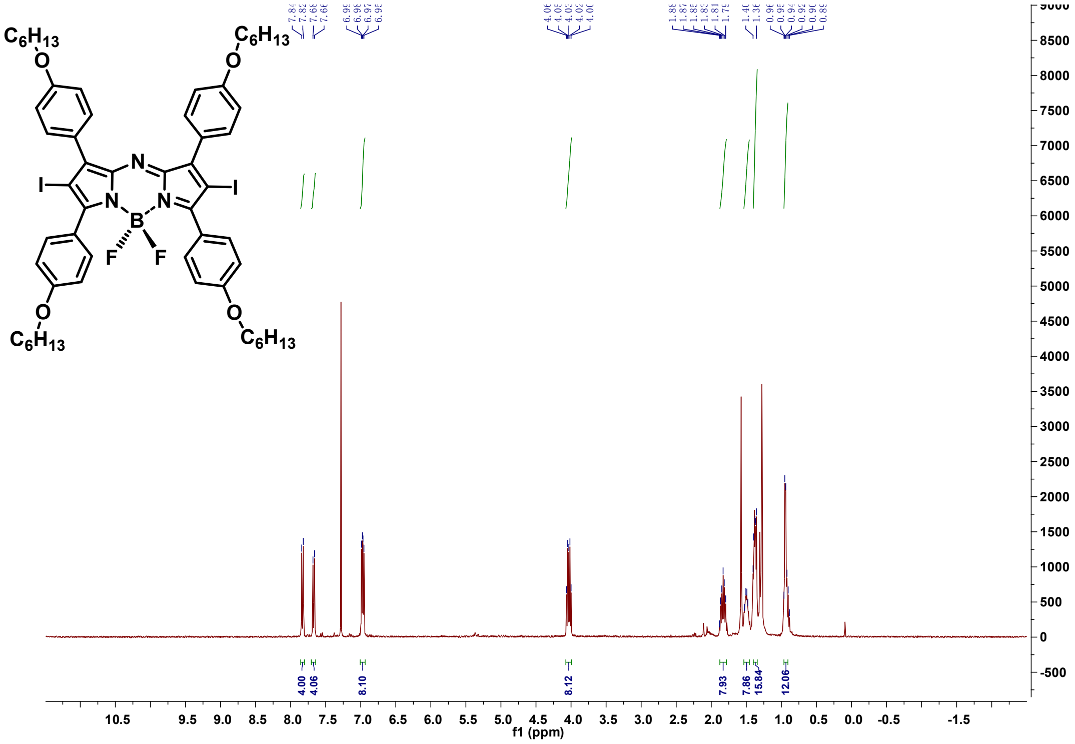

^{13}C NMR spectrum of B1

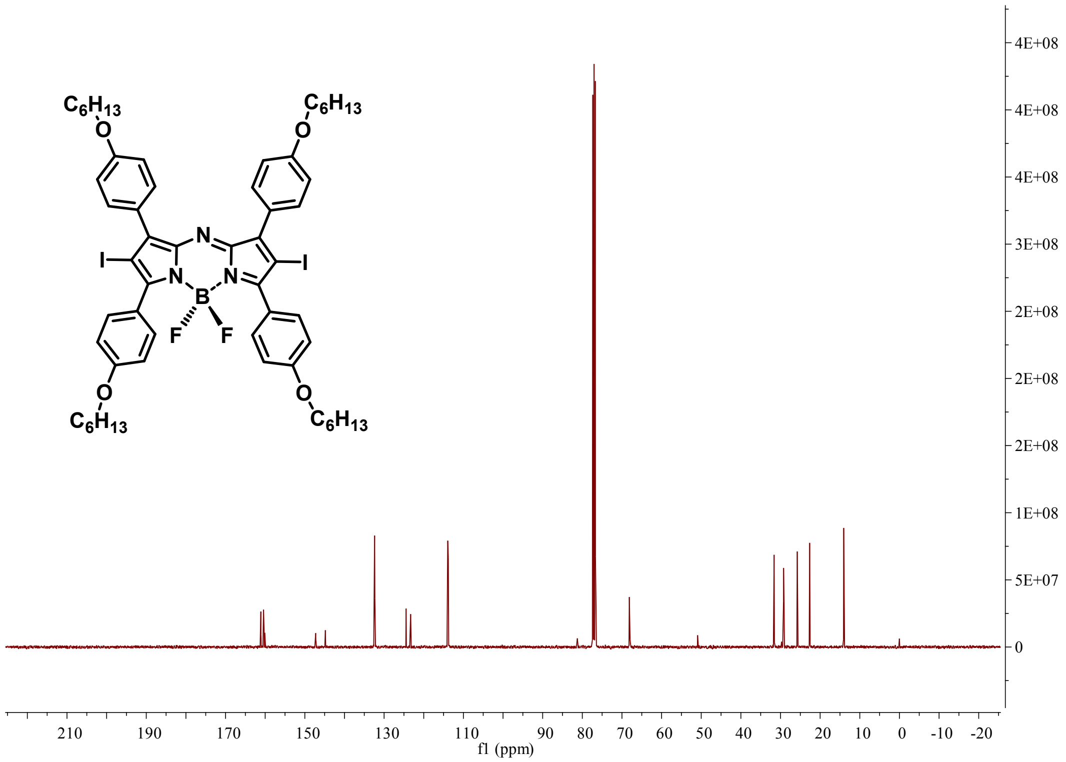

MALIDI-TOF-MS spectrum of B1

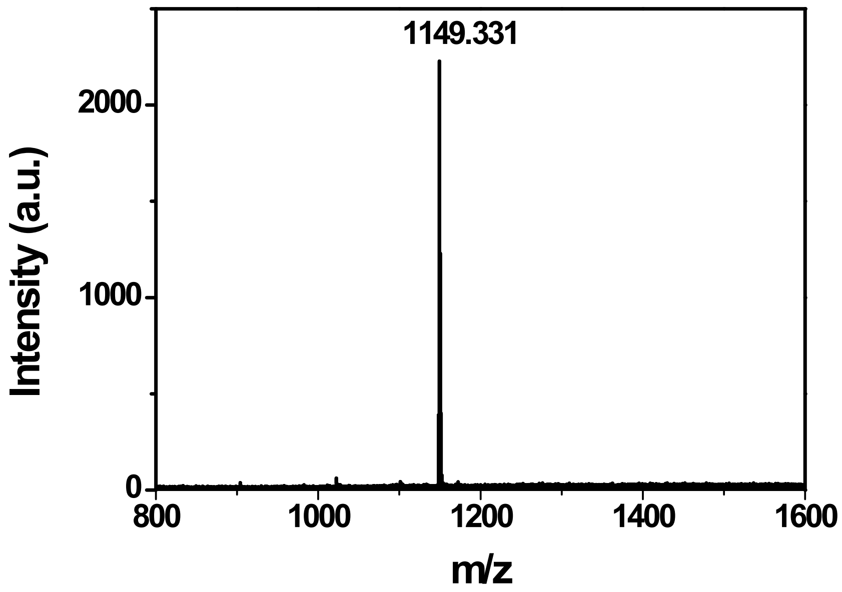

Reference

1. Zhao M., Xu Y., Xie M., Zou L., Wang Z., Liu S., Zhao Q., *Adv. Healthcare Mater.*, 2018, **18**, 1800606.
2. Khodaveisi J., Banejad H., Afkhami A., Olyaie E., Lashgari S., Dashti R. *J. Hazard Mater.*, 2011, **192**, 1437.
